# Phenotypic transformation of HCC827 cells revealed by evaluation of human platelet lysate as a sustainable fetal bovine serum replacement

**DOI:** 10.3389/fcell.2026.1791121

**Published:** 2026-05-13

**Authors:** Clemens Woitaske-Proske, Niklas Keller, Louisa Zinke, Dennis Schade, Christian Peifer

**Affiliations:** 1 Department of Pharmaceutical Chemistry, Christian-Albrechts-University, Kiel, Germany; 2 DZHK, German Center for Cardiovascular Research, Kiel, Germany

**Keywords:** EMT, fetal bovine serum, HCC827, human platelet lysate, integrin signaling, kinomics, NSCLC, sustainability

## Abstract

Fetal bovine serum (FBS), a widely used supplement in cell culture, raises ethical and scientific concerns due to its animal origin, batch variability, and limited physiological relevance for human cells. As part of our efforts to adopt more sustainable and human-relevant cell culture conditions, we investigated human platelet lysate (HPL) as an alternative to FBS for culturing non-small cell lung carcinoma (NSCLC) cells. Different compositions of both sera were assessed by ELISA, showing comparable FGF2 content but 2-fold higher amount of TGF-β1 in HPL compared to FBS. Notably, when cultured with HPL, HCC827 cells developed distinct phenotypes, including ring-like f-actin structures, increased spheroid roundness and size. Canonical endothelial-to-mesenchymal transition (EMT) was not detected, supported by Western blot analysis of key markers Vimentin and SNAI1. Instead, a hybrid EMT signaling state based on kinome activity profiling data is visible but cannot fully explain the visible phenotypical changes. Furthermore, kinome activity profiling at different timings revealed significant HPL-dependent changes for HCC827 cells pointing at altered integrin signaling, distinct from those observed in the other NSCLC lines A549 and H1299. Our findings highlight the cell-type-specific effects of HPL compared to FBS and underscore the importance of case-by-case evaluation when considering HPL as an alternative to FBS in cellular models.

## 1 Introduction

Fetal bovine serum (FBS) is the most commonly used adherent cell culture supplement as it provides essential growth factors, hormones, vitamins and nutrition that promote cell growth and cellular attachment. Yet, derived from unborn cattle, FBS raises ethical concerns over the slaughter of pregnant cows and subsequent serum extraction from live fetuses. Given the wide usage, it also contributes to environmental burdens due to intensive cattle farming as a driver of climate change. The utilisation of FBS and search for alternatives in cell culture is therefore linked to the United Nations’ Sustainable Development Goals (SDGs) of responsible consumption and production (SDG 12), as well as to good health and wellbeing (SDG 3). Further scientific related challenges include batch variability and variable impacts on genotypic and phenotypic effects in cellular models (Johnson and Johnson, 1995; Jochems et al., 2002). Cell cultures are an indispensable *in-vitro* tool, for pre-clinical research, particularly in the context of cancer biology. Traditionally, two dimensional cultures have dominated this field due to their practicability and compatibility with high-throughput screening methods. However, such cellular models are limited in their morphological relevance, e.g., regarding cell-cell or cell-matrix interactions, often not reflecting an *in-vivo* tumor environment (Kapałczyńska et al., 2018). Three-dimensional cell cultures have been developed to overcome those shortcomings. These models like spheroids, first developed in 1970, or more complex organoid systems allow researchers for more meaningful *in-vitro* experiments that better reflect *in-vivo* situations (Costa et al., 2016; Moro et al., 2024; Mangani et al., 2025).

Moreover, culturing human cells with cattle-derived FBS raises the question of *in-vivo* physiological relevance when used, e.g., as cellular *in-vitro* models in drug discovery projects. Owing to these issues, studies are performed to provide meaningful alternatives for FBS as the current standard nutrition supplement in cell culture media. An option is human platelet lysate (HPL), derived from expired platelet concentrates of human blood bottles. The HPL serum contains a distinct variety of growth factors, minerals, and cytokines, offering more physiological conditions for human cell culture (Burnouf et al., 2016). It has already been successfully implemented as a superior alternative to FBS for specific applications such as the cultivation of mesenchymal stem cells (MSCs) (Shanskii et al., 2013). However, little is known about the specific effects of HPL when culturing adherent cancer cell lines *in-vitro*. Although both FBS and HPL are of mammalian origin, their cytokine compositions have been shown to differ vastly (Burnouf et al., 2016). Owing to these differences, we anticipate, that HPL is capable of inducing distinct cellular responses compared to FBS, including altered phenotypes and variable kinome activity.

To address this hypothesis, we investigated HPL as a serum replacement for the cultivation of non-small-cell lung carcinoma (NSCLC) cell lines. NSCLC make up approximately 84% of all lung cancers and are the leading cause of cancer deaths in the US (Ganti et al., 2021). Among the most commonly used NSCLC cell lines for research and drug development are A549 cells (alveolar basal epithelial cell line derived from pulmonary carcinoma), H1299 cells (epithelial-like cell line), and HCC827 cells (cell line with an EGFR mutant resulting in its constitutive activity). It has been reported that about 15%–20% of all NSCLC involve mutations in the epidermal growth factor receptor (EGFR) leading to enhanced tumor growth, rendering HCC827 especially interesting as model for pre-clinical drug development (Gazdar et al., 2010). Current treatment strategies focusing on EGFR in mutant NSCLC often succumb to resistance mechanisms such as epithelial-to-mesenchymal transitioning (EMT) (Peindl et al., 2022; Tulchinsky et al., 2019). EMT induces alterations in cell polarity and adhesion and is implicated, e.g., in tissue fibrosis, tumor invasiveness, and metastasis. The change from an epithelial to a mesenchymal phenotype goes along with the loss of several markers like E-Cadherin and upregulation of markers like SNAI1, Vimentin and N-Cadherin. In cells, these alterations often result in significant phenotypic and functional transitions.

In this study we report on our attempts to replace FBS by differently composed HPL towards their respective effects on cell viability, kinome activities, and three-dimensional growth in NSCLC cell lines. The results may be applicable to further cellular models.

## 2 Materials and methods

### 2.1 Chemicals and reagents

Pierce Bradford Plus Protein Assay Kit, Pierce™ BCA Protein Assay Kits, MPer lysis buffer, Halt® Phosphatase-Inhibitor-Cocktail and EDTA-free Halt® Protease-Inhibitor-Cocktail were purchased from Thermo Fisher Scientific. Alexa Fluor 555 Phalloidin (A34055) was purchased from Thermo Fisher Scientific. Primary antibody Anti-Vimentin (10366-1-AP) was purchased by Proteintech, anti-E-Cadherin (610182) by BD-BioScience and anti-SNAI1 (C15D3) by Cell Signaling Technology. Secondary antibody goat-anti-rabbit HRP was purchased from abcam and goat-anti-mouse HRP was purchased from Proteintech. PTK and STK PamChip4 with reagent kits were purchased from PamGene. Human TGF-β1 ELISA Kit and Human FGF2 ELISA Kit were bought from Proteintech. TGF-β1 was purchased from PeproTech. Well plates and culture dishes were purchased from Sarstedt. RIPA buffer was produced in-house (25 mM Tris-HCl, pH 7.5, 150 mM NaCl, 10 mM sodium cholate, 1% NP-40). Promega Dual Luciferase Assay Kit was purchased from Promega. Lipofectamine 2000 was purchased from Thermo Fisher. Spheroid culture plate was purchased from faCellitate (BIOFLOAT 96 well plate).

### 2.2 Cell lines and cell culture

Non-small cell lung cancer cell line HCC827 was obtained from Leibniz Institute DSMZ; A549, H1299 and HEK293T cells were obtained from ATCC. HCC827 and H1299 were cultured in RPMI1640 (Gibco; Thermo Fisher Scientific, Inc) media supplemented with 10% FBS (Gibco; Thermo Fisher Scientific, Inc) or 10% HPL (PLBioScience), respectively, as well as GlutaMAX (Thermo Fisher Scientific). A549 were cultured in F12K media (Gibco; Thermo Fisher Scientific, Inc) supplemented with 10% FBS or 10% HPL. HEK293T cells were cultivated in DMEM media (PanBiotech) supplemented with 10% FBS. Cells were grown at 37 °C in a humidified 5% CO_2_ atmosphere and grown to a maximum of 80% confluency before splitting. Cell cultures are routinely checked for *mycoplasma* contamination.

### 2.3 Kinase activity screening (PamGene®) and BioNavigator63® analysis

Each cell line was divided into four groups. Cells in group 1 and 2 were maintained in media containing 10% FBS or 10% HPL for 5 days with last media change 2 days prior to lysis and lysed according to the manufacturers protocol after reaching 80% confluency in 100 mm cell culture dishes. These conditions are further called “lasting” conditions. Group 3 and 4 were grown on 10% FBS and 10% HPL for 5 days, respectively, until reaching 80% confluency in 100 mm cell culture dishes. 10% FBS or 10% HPL was added for 30 min prior to lysis. These conditions are further called “fresh” conditions. Cell lysis was based on the manufacturers protocol (PamGene Protocol 1160) with 75 μL M-PER^TM^ (Thermo Scientific) spiked with 1:100 Halt® Phosphatase-Inhibitor-Cocktail (Thermo Scientific) and 1:100 EDTA-free Halt® Protease-Inhibitor-Cocktail (Thermo Fisher Scientific). Protein concentrations of the samples were evaluated using the Pierce Bradford Plus Protein Assay Kit (Thermo Fisher Scientific). Kinase activity profiling was performed using Tyrosine-Kinase and Serine-/Threonine-Kinase PamChip-4® Arrays (PamGene) using a PamStation® 12. Each chip contains four arrays consisting of porous silica. 196 (PTK) or 144 (STK) distinct peptide targets, composed of 13 amino acids mimicking phosphorylation sites, are printed in spots on the porous silica surface. For tyrosine-kinase analysis, FITC-conjugated antibody was used to quantify the phosphorylation level. Furthermore, the kinetic of each reaction was measured by pumping the sample fluid through the porous surface for 94 cycles. From cycle 37 to 92 every five cycles and at cycle 94 five pictures each were taken with different exposure times (10 ms, 20 ms, 50 ms, 100 ms and 200 ms). For serine-threonine kinase analysis, a primary antibody was used and after cycle 90 a secondary FITC-antibody was raised against the primary one for quantification. From cycle 92 to 122 every five cycles and after cycle 124 five pictures each with different exposure times (10 ms, 20 ms, 50 ms, 100 ms and 200 ms) were taken. The brightness of each spot was measured against reference spots and used for further analysis. The kinase activity profiling was performed using 2 independent biological replicates distributed each on different chips and arrays with 5 µg (PTK) or 1 µg (STK) protein per array and a final concentration of 400 µM ATP according to the manufacturers protocol. BioNavigator63® (PamGene) was used for signal evaluation and upstream kinase analysis following the manufacturers protocol. Each condition was prepared in duplicates and means calculated using the BioNavigator63® software from PamGene. Besides this calculation, a clustered heatmap was created. Furthermore, BioNavigator was used for upstream kinase analysis, as the software is able to give a prediction of most increased/decreased active kinases out of the phosphorylation pattern on the peptide chip if two treatments are compared.

### 2.4 ShinyGO enrichment and pathway analysis

Results of upstream kinase analysis from PamStation assays were used for further pathway analysis. Therefore, kinases with increased activity (threshold cut-off at < 1.2 median final score was made according to the manufacturers recommendations) were translated into their corresponding genes using the UniProt database and analyzed using ShinyGO v0.741 for enrichment. Fold enrichment and number of related genes were used for threshold cut off. Using the ShinyGO algorithm, Gene Ontology pathway terms, based on annotations from Ensembl and STRING-db, were used for further depiction and enrichment (Aleksander et al., 2023; Ashburner et al., 2000; Dyer et al., 2025; Mering et al., 2005).

### 2.5 ELISA growth factor assays

For comparison of growth factor concentrations in FBS and HPL, ELISAs specific for FGF2 and TGF-β1 were performed according to manufacturer’s instructions. Sample dilutions were 1:1, 1:2, and 1:4. Using polynomial integration, standard curves were calculated and used for determination of unknown concentrations in HPL and FBS.

### 2.6 TGF-β/smad reporter gene assay

HEK293T cells were co-transfected in bulk with an SBE4-Luciferase and TK-driven *Renilla* luciferase plasmid as previously described (Längle et al., 2024; Schade et al., 2012). Briefly, 20000 cells were plated per well in a 96 well plate in DMEM media and treated with either TGF-β1 (0–20 ng/mL), HPL or FBS (0%–20%) for 22 h. Each condition was carried out in technical triplicate. After treatment, the media was aspirated, and cells were lysed according to the manufacturer’s protocol (Promega Dual Luciferase Assay Kit).

### 2.7 Bright-field microscopy

30000 cells were seeded into each well of a 6-well-plate and 10000 cells were seeded into each well of a 12-well plate, respectively. Images were acquired using the ECHO Rebel microscope using ×10 and ×4 magnification.

### 2.8 Immunostaining & confocal microscopy

For confocal microscopy analysis HCC827 cells were plated using 30000 cells per well onto cover slips in a 12-well plate and grown to moderate confluency, so that single cells were still visible. Cells were grown in media supplemented with either 10% FBS or HPL added. FBS media was additionally treated with 10 ng/mL TGF-β1. After 3 days cells were fixed using freshly prepared 4% PFA in dPBS solution for 10 min at room temperature using 100 µL per well. Next, cells were permeabilized using a mixture of 0.2% Triton-X 100% and 5% FBS in PBS using 250 µL per well and incubated for 20 min at room temperature. Afterwards, permeabilization buffer was removed and staining buffer containing Alexa Fluor 555 Phalloidin (1:40, Invitrogen) was added and incubated for 2 hours at room temperature. Subsequently, staining buffer was removed, and cells were washed three times using 500 µL PBS. Last, cells were stained with Hoechst33342 for 10 min using a 1:1000 dilution before washing another three times with dPBS. Confocal images were generated using the Zeiss LSM 900 confocal-laser-scanning microscope with AiryScan2.

### 2.9 Immunoblotting

Treated cells were washed with 1 × dPBS, lysed in RIPA lysis buffer, supplemented with a 1:1 mixture of 1 mM phenylmethylsulfonyl fluoride (PMSF) and 1 mM pepstatin A solution as well as 2 mM NaF and 10 mM Na_2_P_4_O_7_, and centrifuged (12000 g, 4 °C, 15 min). Protein concentrations were measured using the Pierce™ BCA Protein Assay Kits. Lysates were prepared and separated in 10% sodium dodecyl sulphate polyacrylamide gel electrophoresis (SDS-PAGE) gels with 20 µg protein per lane. Proteins were transferred to nitrocellulose (SNAI1) or PVDF (E-cadherin, Vimentin) membranes and blocked with 5% skim milk at room temperature for 1 h with constant 200 mA. Membranes were exposed to the following primary antibodies overnight, at 4 °C: anti-Vimentin, anti-E-Cadherin and anti-SNAI1. All primary antibodies were used at 1:1.000 dilution, unless specified otherwise. The following day, membranes were washed with Tris-buffered saline with added Tween-20 (TBST) and exposed to either goat-anti-rabbit or goat-anti-mouse HRP-conjugated secondary antibodies for 1 h at room temperature. Membranes were washed with TBST and imaged via Clarity Max Western ECL Substrate on a ChemoStar Touch ECL & Fluorescence Imager.

### 2.10 Spheroid cell culture

HCC827 cells were grown in RPMI1640 media supplemented with FBS or HPL for 1 week to reach 80% confluency. Afterwards, cells were seeded into BIOFLOAT 96 well plate with 150 µL media at seeding density of 10000 cells/well. Cells were grown for 4 days. Spheroids were imaged using an ECHO Rebel microscope using ×10 magnification. Images were then analyzed through a CellProfiler pipeline, identifying the main spheroid and calculating the area as well as the maximal ferret area and the minimal ferret area. The ferret roundness was then calculated by diving the minimal area by the maximal area.

### 2.11 Cell viability (resazurin) assay

Cell viability and reductive capacity was measured using a resazurin assay. 30000 cells were plated into 96-well plates containing 200 µL RPMI1640 media supplemented with 10% FBS. After attaching over 24 h, media was changed to 1%, 5% and 10% FBS or HPL, respectively. Cells were incubated over 48 h at 37 °C in humidified atmosphere with 5% CO_2_. Next, media was removed and 120 µL of 0.00167% resazurin in dPBS was added and incubated for 2 h at 37 °C. Absorption was measured with excitation at 544 nm and emission at 590 nm using a Tecan Spark Multimode Microplate Reader.

### 2.12 Cell growth assay

For measurement of cell growth over time, 10000 cells were plated into four 12-well plates containing 2 mL of RPMI1640 media supplemented with 10% FBS. Cells attached overnight, before media was removed and two 12-well plates were filled with RPMI1640 media supplemented with 10% FBS and other two 12-well plates with RPMI1640 media supplemented with 10% HPL. Cells were washed three times with dPBS, fixed in triplicates at defined time points using freshly prepared 4% PFA in dPBS solution for 10 min at room temperature using 500 µL per well and again washed three times with dPBS. 500 μL dPBS was left in wells against drying out. After 96 h when all wells were fixed, cells were permeabilized using a mixture of 0.2% Triton-X 100% and 5% FBS in dPBS using 250 µL per well and incubated for 20 min at room temperature. Afterwards, cells were stained using Hoechst33342 for 10 min using a 1:1000 dilution before washing another three times. Measurements of nuclei counts were conducted using an ImageXPress® Micro Confocal High-Content Imaging System and images were analyzed using Cell Profiler.

## 3 Results

### 3.1 NSCLC cultured in HPL or FBS: phenotypical changes, viability and doubling time

**FIGURE 1:**
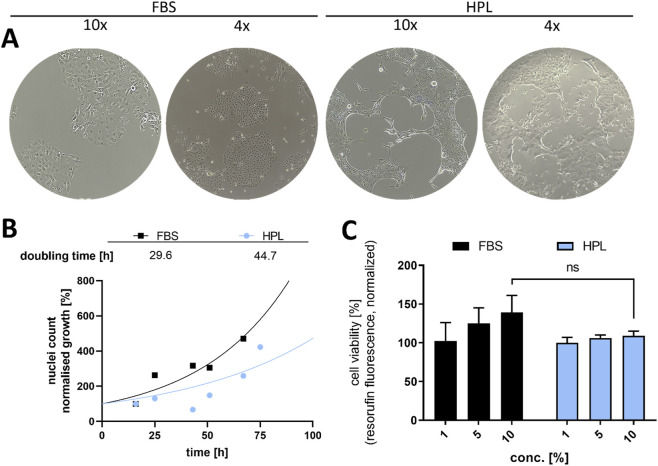
Comparison of HCC827 cells cultured in fetal bovine serum (FBS) vs. human platelet lysate (HPL). **(A)** Representative brightfield images demonstrating cell morphology in media containing 10% FBS or 10% HPL. Magnifications are ×4 and ×10 (Further images in Supplementary Figures 1–7). **(B)** Proliferation curves of HCC827 cells in 10% FBS or 10% HPL, tracked via relative nuclei count over 76 h **(C)** Dose-response analysis of cell viability, measured by a resazurin-based metabolic assay (resorufin fluorescence, λex/em 544/590 nm), in media supplemented with 1%–10% of either FBS or HPL.

To assess the differences between HPL and FBS on the cellular phenotype, the NSCLC cell lines A549, H1299 and HCC827 were either cultured with 10% FBS or 10% HPL. In HCC827 cells, HPL-cultured cells exhibited distinct morphological differences after 5 days when compared to those of FBS treatment (Figure 1). In contrast to HCC827 cells, significant morphological or phenotypical changes could not be observed in the NSCLC cell lines A549 and H1299. However, HCC827 cells grown with HPL display enhanced light-scattering around the cellular outlines, indicating increased complexity with multicellular structures and enhanced edge thickness. Furthermore, HCC827 cells showed significant reorganization, forming gaps between cell clusters, a feature that is consistent with cytoskeletal architecture changes. Additionally, single cells exhibited a stretched phenotype and increased cellular mobility. To determine whether the morphological changes in HCC827 correlated with differences in cell proliferation and viability, we assessed their growth kinetics and metabolic activity in more detail. Cellular growth of HCC827 influenced by either HPL or FBS was analyzed by culturing cells in a 12-well plate over a period of 76 h, respectively (Figure 1). In general, cells grown with HPL displayed slower growth when compared to cells grown with FBS. The metabolic activity and overall viability of HCC827 cells showed no significant differences between 1% and 10% HPL, indicating saturation of cellular requirements already at low concentrations of HPL. Conversely FBS-cultured cells displayed a more concentration-dependent increase in metabolic activity, indicative of progressive saturation with increasing serum content. These results demonstrate a different phenotype of HCC827 cells grown with supplementation of HPL instead of FBS, correlating with significantly decreased doubling time and metabolic activity. In total, HCC827 cells in HPL maintained a lower viability and proliferation, whilst displaying a notably different cellular organization and clustering, indicating a difference in cellular homeostasis compared to FBS conditions.

### 3.2 Multiplexed kinase activity screening

**FIGURE 2:**
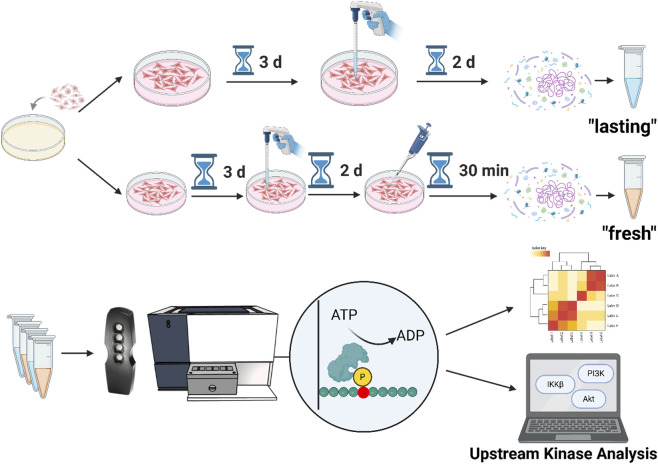
Schematic overview of the comparative kinome profiling workflow. Three NSCLC cell lines (A549, H1299, and HCC827) were treated with media supplemented with either 10% HPL or 10% FBS. Two treatment timelines were investigated: (1) a long-term (“lasting”) exposure for 5 days, with media replenished after day three; and (2) a short-term (“fresh”) stimulation for 30 min, performed on the fifth day of culture. Following treatment, cell lysates were collected and analyzed on PamChips using the PamStation platform. Phosphorylation patterns were quantified using BioNavigator63 software, and further investigation was conducted via upstream kinase analysis (Figure created with BioRender.com). The respective kinome activities of A549 and H1299 serve as suitable background controls when compared to HCC827 (peptide phosphorylation heatmap in Supplementary Figures 10, 11).

As there was no clear starting point for investigating in detail what causes the visible phenotypical and morphological changes particularly in HCC827 cells, we initially conducted multiplexed kinase activity profiling using the PamStation® system. Alterations in kinomics can reveal detailed information on processes within the cells (Manning et al., 2002; Greenman et al., 2007; Shchemelinin et al., 2006). To assess kinome activities, we treated the different NSCLC lines A549, H1299 and HCC827 under four distinct conditions and respectively measured peptide phosphorylation levels using protein tyrosine kinase (PTK) and serine-threonine kinase (STK) arrays. Hence, we cultured the cell lines for 5 days with 10% HPL or 10% FBS serum and then either lysed them directly (“lasting” condition) or subjected them to a 30-min stimulation (“fresh” condition) with the same supplement. We selected the “fresh” 30-min timepoint based on previously reported receptor activation kinetics (Wang et al., 2017), while the “lasting” condition was chosen to capture downstream, likely transcriptionally mediated, effects that typically emerge after several hours of stimulation (Liu et al., 2023). These procedures enabled us to interrogate both sustained signaling and early membrane receptor-driven kinase responses which we assessed by upstream kinase and pathway analysis with the BioNavigator63 analysis tool from PamGene (Figure 2).

**FIGURE 3:**
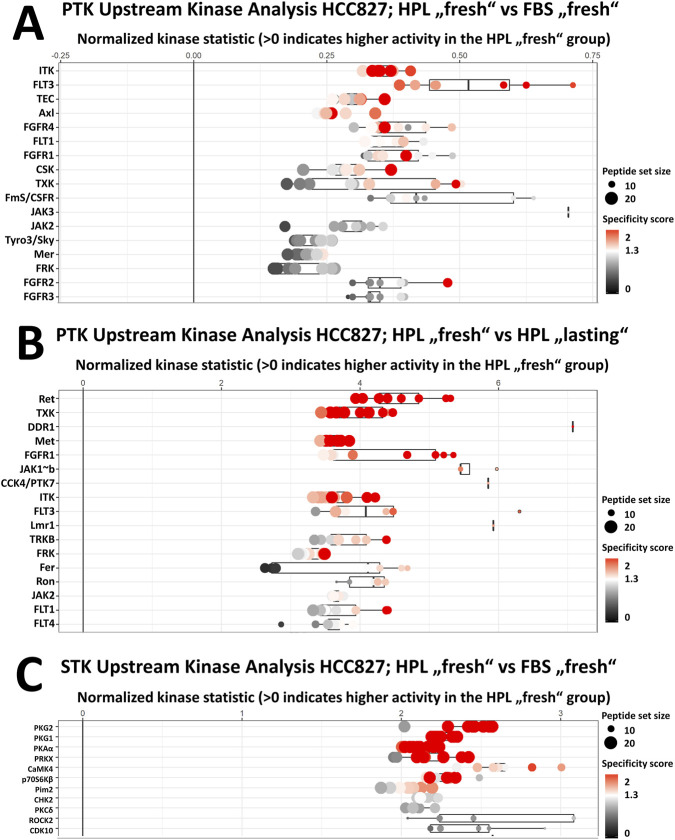
Upstream kinase analysis (UKA) in HCC827 cells depending on HPL compared to FBS supplementation. For creation of an UKA, an algorithm from the manufacturer PamGene uses the peptide phosphorylation pattern on chip to infer whether specific kinases that phosphorylate the specific peptides on chip are increased or decreased active comparing two conditions. Increased active kinases have a positive value on the x-axis, which displays the log fold change in kinase activity. Single dots for one kinase represent the phosphorylation of the peptides on chip referring to this kinase. Size of the dots represent the overall peptide set size on chip, e.g., how many peptides on chip refer to this specific kinase. Last, the color of these dots refers to a specificity score which is based on permutation tests to show the probability that the observed effect is correlated to the peptide set and can not be obtained using a random set of peptides. For further information, please visit the manufacturers information sheet (PamAcademy, https://pamgene.com/wp-content/uploads/2024/08/Flyer_PamGene-Upstream-Kinase-Analysis-Tool.pdf). Plots display **(A)** the most increased active tyrosine kinases for the comparison of HCC827 cells freshly activated with HPL or FBS and **(B)** the most increased active tyrosine kinases for the comparison of HCC827 cells freshly activated with HPL or lasting activation with HPL, predicted from their specific peptide phosphorylation patterns. **(C)** Plot displays the most increased active serine-threonine kinases for the comparison of HCC827 cells freshly activated with HPL or FBS. Values higher than one indicate higher activity of kinases shown. Not all kinases from analysis are shown as PamGene recommends a threshold cut of < 1.2 for median final score (specificity score). Full result chart as well as comparison of serine-threonine kinases for the comparison of freshly activated HPL with lasting activation with HPL can be seen in Supplementary Figures 8, 9. Size of dots stands for peptide set size on chip used for specific kinase activity calculation.

Interestingly, we detected distinct kinome activity patterns for each condition in every cell line, but only minor differences in A549 and H1299, further supporting the above described phenotypical findings. In contrast to A549 and H1299, the HCC827 cells exhibited pronounced differences across conditions, indicating a cell-line-specific response to serum replacement (Burnouf et al., 2016). To analyze these changes in more detail, we performed upstream kinase analysis for HCC827 using both STK and PTK chips (Figure 3 and Supplementary Figures 8, 9) and compared the results with published data to rationalize the distinct phenotype.

**FIGURE 4:**
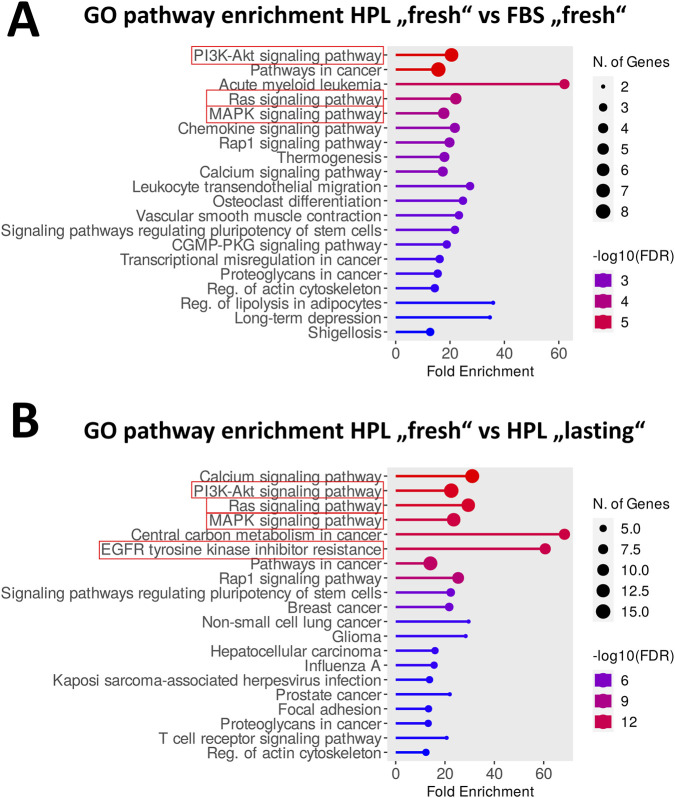
Pathway enrichment analysis show critical differences in PI3K/Akt-, Ras- and MAPK-signaling networks through “fresh” activation with HPL. **(A)** Translation of most increased active tyrosine and serine-threonine kinases (threshold cut of < 1.2 for median final score) into correlating genes using UniProt database, ShinyGO v0.74 for pathway enrichment yields most affected pathways between treatment condition of “fresh” HPL activation compared to “fresh” FBS activation. **(B)** Pathway enrichment for corresponding genes to most increased active kinases for comparison of “fresh” with “lasting” HPL treatment. Dot size stands for number of related genes and color for -log10 of false discovery rate (FDR). Signal pathways framed in red boxes point to the later proposed hypotheses.

For pathway enrichment analysis (Figure 4), we mapped the genes corresponding to highly active kinases via the UniProt database and analyzed GO pathway terms for enrichment using the ShinyGO algorithm (The UniProt Consortium et al., 2025; Ge et al., 2020).

**FIGURE 5:**
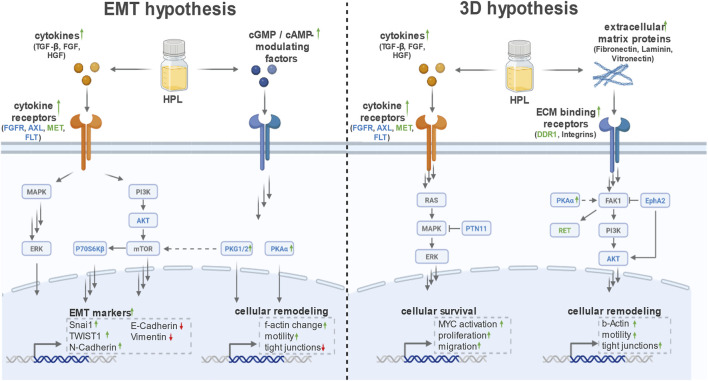
Analysis of multiplexed kinase activity screening suggested two hypotheses of an occurring endothelial-to-mesenchymal transition (EMT) or increased 3D growth through activation of integrin signaling. Kinases with increased activity and peptides with increased phosphorylation are colored in blue and green (Figure created with BioRender.com).

The kinome-based evaluation of the HPL triggered signaling effects in HCC827 cells suggested two different hypotheses: 1) an occurring endothelial-to-mesenchymal transition (EMT) or 2) enhanced 3D-like growth through activation of integrin signaling (Figure 5).

The EMT hypothesis is based on the observation that “fresh” stimulation via HPL, when compared to FBS, leads to higher activity levels for a plethora of EMT-related kinases (see Figure 3). Namely, for the PTK chips (see full analysis in Supplementary Figure 8) we identified increased activation of all four subtypes of fibroblast growth factor receptors (FGFR), fms-like tyrosine kinase (FLT), and AXL (also known as Tyro3) by the HPL stimulus. All of these kinases have previously been reported to be involved in the EMT process (Brown et al., 2016; Wang et al., 2018; Chen et al., 2020; Bivona and Okimoto, 2015). The same applies to the comparison of “fresh” stimulation to “lasting” stimulation for HPL, but in this case MET (also known as hepatocyte derived growth factor receptor) showed increased activation, converging to drive transcriptional EMT programs. We observed the MET activation only in the comparison of the “fresh” vs. “lasting” condition of HPL, but not for FBS. Interestingly, MET has previously been reported to also be involved in the promotion of an EMT (Lee et al., 2017; Liu et al., 2017).

We next performed the same analysis for the STK array data (see full analysis in Supplementary Figure 9) and observed specific upregulation for PKAα, PKG1 and PKG2 as well as for p70S6Kβ, which collectively reorganize actin dynamics. In line with this notion, the listed PKs are key modulators of cytoskeletal organization and cell motility. The cytoskeletal organization and motility are known prerequisites for a successful EMT. Furthermore, p70S6K is a downstream effector of mTOR and closely connected to another increased active kinase CaMK4 (Xu et al., 2024). This links to PI3K-AKT-mTOR, a pathway that is closely connected to EMT and is thus also an additional indicative factor (Wu et al., 2016). This pathway was also identified to be significantly upregulated within the GO analysis (ShinyGO analysis, see Figure 4). EGFR TKI resistance enrichment reinforces this, as an EMT in HCC827 confers therapy escape (Tulchinsky et al., 2019). Taken together, HPL-stimulation leads to the activation of some EMT-related pathways and kinases. Noteworthy, this effect is however not due to the alteration of a specific kinase but rather based on the symphony of a substantial kinome-wide remodeling in response to the HPL stimulus.

**FIGURE 6:**
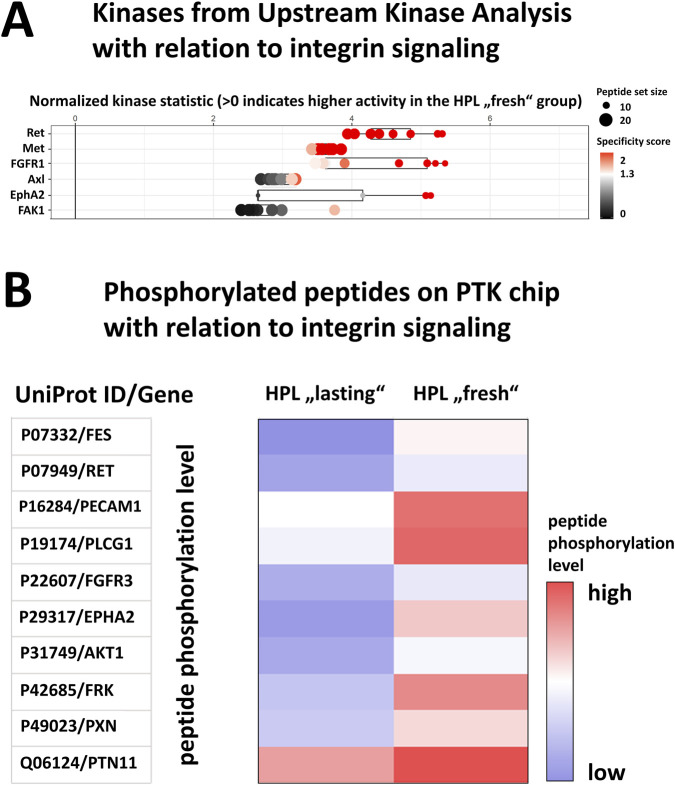
HPL supplementation initially activates integrin signaling in HCC827 cells. **(A)** Upstream Kinase Analysis identifies higher activity of integrin-associated kinases when comparing “fresh” HPL stimulation to “lasting” (specific peptide phosphorylation changes as database for upstream kinase analysis in Supplementary Figures 17–22). **(B)** PTK-PamChip showing increased phosphorylation of integrin-related peptides following fresh HPL activation compared to lasting HPL treatment.

Regarding the observed 3D clustering of HCC827 cells under HPL conditions, we hypothesized that this mechanism may be driven by an extracellular matrix protein induced integrin signaling (Burnouf et al., 2016). Extracellular matrix proteins like fibronectin, vitronectin, and laminin, can attach to and activate integrin receptors on the cell surface. The signal evolves by increasing the activity of focal adhesion kinase (FAK), ultimately leading to the activation of the MAPK pathway that results in the expression of E-Cadherin mediating cell-cell attachments (Smyrek et al., 2019; Lin et al., 2006; Renshaw et al., 1999; Schaller, 2010). This proposed mechanism is supported by the fact that GO enrichment analysis focusing on pathways yielded an increased MAPK pathway activation as well as an increased FGFR1 signaling (Figure 6, consistent with our upstream kinase analysis in Figure 3A) (Mori and Takada, 2013; Miyamoto et al., 1998).

Although upstream FAK1 was not directly detected, downstream evidence confirms robust integrin signaling under fresh HPL: RET (FAK1 target) and EphA2 (which dephosphorylates FAK1) were upregulated, indicating progression to downstream remodeling within the 30 min timeframe chosen within the experimental setup (Miao et al., 2000; Plaza-Menacho et al., 2011). Furthermore, the measured higher activity of c-Met (the HGF receptor) could be based on the fact that HPL has higher concentrations of HGF when compared to FBS, and that integrin interaction leads to activation of c-Met (Burnouf et al., 2016; Stanislovas and Kermorgant, 2022). Axl, one of the proteins that is also an indicator of EMT, is associated with FAK1 activity, explaining why we can see its upregulation in the peptide arrays. A closer look at the integrin-related signaling pathways in the upstream kinase analysis revealed increased activity of tyrosine-protein phosphatase non-receptor type 11 (PTN11 or SHP2). PTN11 is a positive regulator of MAPK signaling and another component of the dephosphorylation of FAK1, interacting further downstream of EphA2. This cascade of FAK1 targets functionally manifests in focal adhesion components: PTN11/SHP2 boosts MAPK propagation, PLCG1 drives fibronectin dependent adhesion, and FAK phosphorylates Paxillin (PXN), a core component of focal adhesions, collectively enabling actin reorganization. FRK links to PI3K/AKT via PTEN stabilization, while Fes and RET promote microtubule assembly (Jones et al., 2005; Tvorogov et al., 2005; Wang et al., 2007; Yim et al., 2009; Plaza-Menacho et al., 2011; Guidetti et al., 2015; López-Colomé et al., 2017). Taken together, these comprehensive kinome activity and peptide phosphorylation data provide strong evidence that the “fresh” stimulation of HCC827 cells with HPL induces a robust activation of integrin signaling pathways, leading to a significant reorganization of the cytoskeleton that can be seen in phenotypical alterations.

Based on these findings, we conclude that the altered signaling environment induced by HPL may 1) engage pathways commonly associated with EMT, potentially contributing to the observed phenotypic changes and increased motility of HCC827 cells, and 2) activates integrin signaling leading to a 3D-like growth. However, results of the multiplexed kinase activity screening are not able to fully explain the observed phenotypic changes or assign the increased active kinases specifically to a hybrid EMT signaling state due to overlapping signaling with integrin signaling pathway activation. To further investigate these conclusions, we performed phenotypic assessments alongside specific analyses of key EMT-associated markers, including f-actin staining as well as Western Blots of E-cadherin, vimentin, and SNAI1.

### 3.3 Composition of several growth factors and EMT hypothesis

**FIGURE 7:**
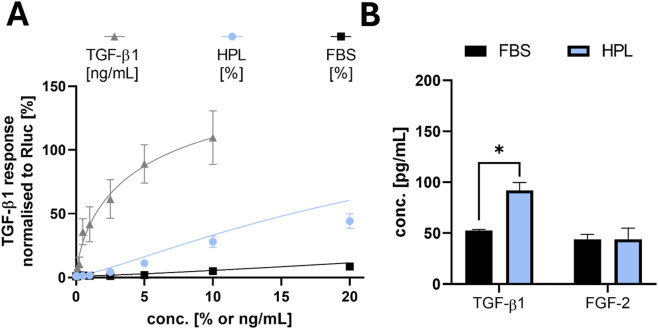
SBE4-based transient dual luciferase reporter gene assay in HEK293T showing HPL/FBS differences in growth factor amounts. In the sera, comparable FGF2 quantities were detected but two-fold increased TGF-β1 concentration in HPL leading to increased TGF-β-receptor response. **(A)** TGF-β1/Smad-reporter gene (SBE4) assay showing TGF-β1 response of HEK293T cells via firefly luciferase signal normalized to Renilla luciferase signal. **(B)** Growth factor concentrations of TGF-β1 (FBS: 53.6 ± 5.0 pg/mL; HPL: 92.0 ± 7.8 pg/mL) and FGF2 (FBS: 44.1 ± 4.6 pg/mL; HPL: 44.1 ± 10.8 pg/mL) quantified by ELISA in FBS (Gibco) and fibrinogen-depleted HPL (PLBioScience). Data is shown as mean ± SEM (n = 3–4), *p ≤ 0.05. For calibration curves of both ELISAs see Supplementary Figure 13.

It has been reported that HPL contains significant amounts of TGF-β and that TGF-β is indicated in the occurrence of an EMT, mainly due to activation of the RhoA/ROCK pathway (Bhowmick et al., 2001). Hence, to investigate the activity of TGF-β in our HPL and FBS supplements, we used a Smad4 Binding Element (SBE4)-based transient dual luciferase reporter gene assay in HEK293T cells. Hereby we observed a 5-fold higher activity of TGF-β in HPL when compared to FBS at 10% serum supplementation, confirming the fact that HPL produces a stronger TGF-β signal in cells (Figure 7).

**FIGURE 8:**
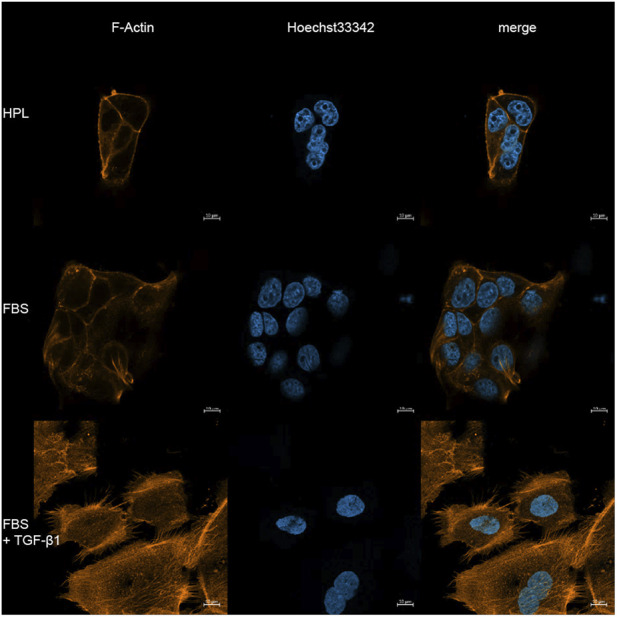
Confocal microscopy of HCC827 cells stained for f-actin (AF555-Phalloidin, red) and nuclei (Hoechst33342, blue). Compared to the punctuate f-actin in FBS controls or the stress fibers induced by TGF-β1 (10 ng/mL), HPL-cultured cells displayed a distinct accumulation of ring-like f-actin structures at cell-cell contacts. Scale bar = 10 µm.

To investigate the underlying mechanisms of the re-organization and metabolic changes in these cells in more detail, further differences in growth factor compositions in both, HPL and FBS should be taken into account. Previous studies found higher amounts of many growth factors in HPL compared to FBS, especially regarding different isoforms of PDGF, TGF-β, FGF, VEGF, BDNF and IGF (Guiotto et al., 2020). Burnouf et al. described discrete ranges of multiple samples of human platelet lysate, but also took a closer look at its global composition. These results show a plethora of significant differences for chemokines, coagulation factors, adhesion molecules, immunogenic molecules, as well as for regulators of cell growth and angiogenesis (Burnouf et al., 2016). Regarding our HPL samples used in this project, EGF and PDGF levels were described by the manufacturer as 1.4 ng/mL and 23.4 ng/mL, respectively. Due to the increased mobility, various notes in scientific literature and our hypothesis of an occurring EMT, we aimed to have a closer look at the interplay between TGF-β and FGF2. The relation of these two growth factors has been previously described being able to induce an EMT or not, depending on their ratio (Santhana Kumar et al., 2018). HCC827 cells were previously reported to undergo EMT when co-treated with FGF2 and TGF-β1, due to the capability of TGF-β1 to induce an isoswitch from FGFR isoform IIIb to IIIc, leading to higher sensitivity for the ligand (Shirakihara, 2011). Based on this context, we conducted ELISAs of TGF-β1 and FGF2, actually showing similar amounts of FGF2 but 2-fold higher amounts of TGF-β1 in HPL compared to FBS. These findings align well with the reporter gene assay results (see Figure 7). We next conducted f-actin staining experiments to see if EMT markers like long strands of f-actin are visible in HPL treated cells (Figure 8). However, to our surprise we could see neither the typical TGF-β induced long strands of f-actin, nor could we detect the FBS cultured typical puncta. Instead, we detected a different phenotype showing high accumulation of ring-like f-actin structures around the cellular contact areas as well as more compact, bean-shaped nuclei in HPL supplemented HCC827 cells. Taken together, the measured increase of TGF-β activity may be not sufficient to induce the typical canonical EMT phenotype but rather points to an epithelial phenotype of the cells. More compact nuclei could be explained by differences in stretch forces on the nuclei. As HPL supplemented HCC827 show morphological differences, changes in chromatin with increased nuclear strain stiffening could explain the more compact nuclei (Bergamaschi et al., 2024). Furthermore, studies have shown that changes in chromation organization can activate genes and following protein transcription and vis versa (Corato and Gomez-Benito, 2024). Therefore, changes in chromatin states and distribution could explain the differences in the morphology of the nucleus.

**FIGURE 9:**
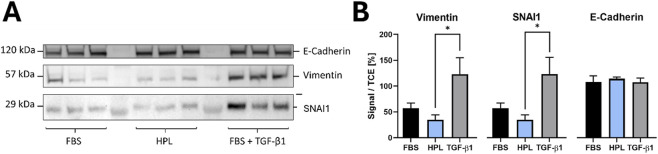
**(A)** Representative Western blots for Vimentin, SNAI1, and E-Cadherin in HCC827 cells cultured with FBS, HPL, or FBS + TGF-β1 as positive control (full-length uncropped blots/gels are presented in Supplementary Figures 14–16) **(B)** Semi-quantitative representation of protein bands (TCE-normalized band intensities). While the TGF-β1 control validated the EMT induction pathway (increased Vimentin and SNAI1), HPL supplementation had the opposite effect, significantly decreasing both mesenchymal markers relative to the FBS control. No significant downregulation of E-Cadherin was observed, even in the positive control. This indicates HPL does not induce a canonical EMT and may instead stabilize an epithelial phenotype. Data is shown as mean ± SD (n = 3), *p < 0.05.

To provide further evidence regarding the missing EMT phenotype (missing long strands of f-actin), we conducted Western blot experiments for EMT key markers Vimentin, SNAI1 and E-Cadherin (Figure 9). Interestingly, levels of Vimentin and SNAI1 were lower in HPL-treated compared to FBS-treated cells. As anticipated, the positive control (FBS + TGF-β1) induced a clear EMT-like response, characterized by increased expression of Vimentin and SNAI1. Compared to the positive control, HPL as well as FBS showed significant lower levels of mesenchymal markers, Vimentin and SNAI1. Even in TGF-β1 treated cells no strong decrease of E-Cadherin was visible. The observed epithelial behavior of HCC827 correlates with reports showing that even in prolonged treatment with TGF-β1 a subpopulation of approx. 30%–40% of the cells remained E-Cadherin positive (Wilson et al., 2014).

Cells cultured with HPL displayed an opposite pattern to the TGF-β1-induced EMT, with slight upregulation of epithelial and downregulation of mesenchymal markers. These results contradicted our initial hypothesis that the visible phenotypical changes are related to HPL leading to an EMT. Based on our kinome activity screening results and visible EMT-markers in Western blot analyses, we suggest a partial or hybrid signaling state of EMT-associated kinases which is leading to the conclusion that HPL-treated HCC827 cells do not undergo a canonical EMT.

### 3.4 Hypothesis of promoted cellular reorganization and 3D-like growth

As observed by initial bright-field microscopy, HCC827 cells cultured in HPL-supplemented media formed distinct cell clusters, suggesting a shift towards 3D aggregation. This observation supports the conclusion that a rich composition of human-derived growth factors and adhesion molecules in HPL may promote this cellular reorganization by fostering a more complex extracellular matrix (ECM) environment when compared to FBS. This hypothesis was corroborated by our prior PamStation data, which indicated the activation of integrin signaling that is typically activated by fibrinogen. Even though we used fibrinogen-depleted HPL to avoid coagulation, there are still significant amounts of ECM-proteins like fibronectin and vitronectin present in HPL (Laurens et al., 2006; Tragoonlugkana et al., 2024). Furthermore, multiple integrins bind to these still existing ECM-proteins like α5β1 to fibronectin and αvβ3 to vitronectin (Hynes, 2002; Horton, 1997). Especially fibronectin induced integrin clustering leads to activation of FAK and its downstream kinases as well as enhancing PI3K/Akt and MAPK signaling, all visible in our previous analysis (Figures 3, 4). Last, especially EGFR mutated NSCLC lines show enhanced growth and survival signaling upon integrin activation through ECM proteins (Wu et al., 2023; Hassanein et al., 2021). Taken together, even fibrinogen depleted HPL contains multiple ECM-proteins which are able to activate integrin signaling in a fibrinogen-like manner.

**FIGURE 10:**
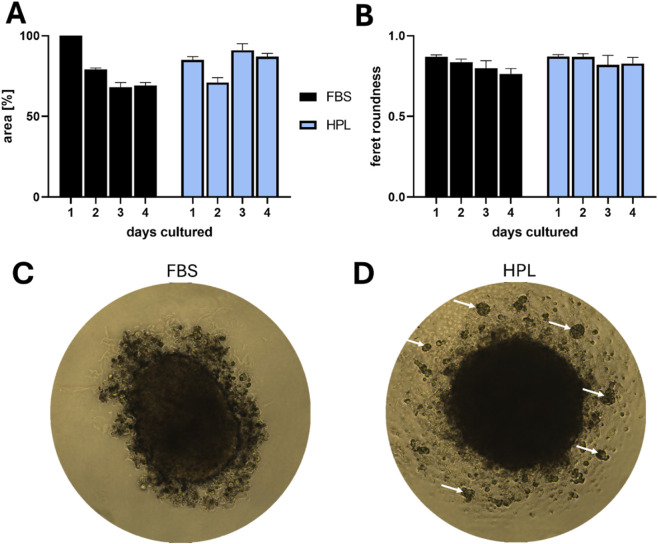
HCC827 spheroids supplemented with 10% FBS or 10% HPL in a BioFloat 96-well plate. **(A)** Area of spheroids was measured, showing that area of FBS-supplemented spheroids shrinks over time while HPL-supplemented spheroids kept their size. **(B)** HPL spheroids also maintain a superior and more constant roundness (Feret diameter) compared to FBS spheroids, which become progressively oval **(C,D)**. Representative images on day four highlight the morphological differences: the FBS spheroid is oval and appears to be dissociating, while the HPL spheroid is round, compact, and is forming secondary satellite spheroids (white arrows), consistent with a stronger cell aggregation-promoting phenotype.

To further investigate the described 3D growth phenotype, we adapted HCC827 cells to media supplemented with either 10% HPL or 10% FBS for 2 weeks. Spheroid growth and roundness were then monitored over 4 days. Herein, spheroids cultured in HPL-supplemented media achieved a more consistent degree of roundness than those grown in FBS (Figure 10). Furthermore, we observed in the HPL group the generation of smaller secondary satellite spheroids after several days of culture, a phenomenon that we could not see in the FBS group.

Interestingly, Zanoni kursiv have shown that volume and shape of spheroids affects their viability. Spheroid shape is connected to core dimension, surrounding shell thickness which contains proliferative cells and therefore also general viability. Non-spherical spheroids reduced distance between single cells and culture media, leading to increased viability through a higher number of active cells (Zanoni et al., 2016; Zhu et al., 2025). As the diameter and roundness as well as satellite spheroids differ between FBS and HPL grown spheroids, a general conclusion on the viability could not be taken as comparisons between this two morphologically different groups would be misleading.

## 4 Conclusion

**FIGURE 11:**
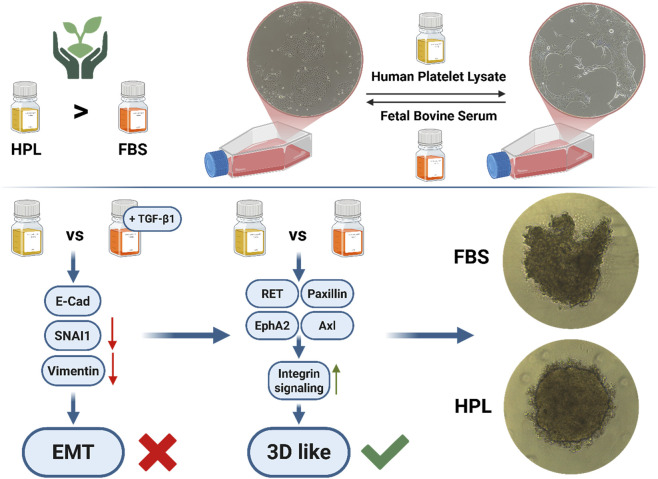
Schematic summary of FBS and HPL induced effects towards alterations in HCC827 cells. Kinase screening and Western blot analysis revealed that HPL promotes a 3D-like growth phenotype, characterized by increased spheroid roundness and driven by integrin-related signaling, rather than a canonical endothelial-to-mesenchymal transition (EMT), leading to the conclusion that characterization of single cell lines is needed to decide if serum replacement works out for the specific research question and lab conditions (Figure created with BioRender.com).

In this study we investigated the effects of HPL compared to FBS in NSCLC cell culture. Our findings present critical insights into the cellular mechanisms driven by HPL, particularly its ability to activate pathways associated with changes in the cytoskeleton, increased cell-cell adhesion and hallmarks of three-dimensional growth in cell culture flasks. Our results revealed that, compared to FBS, supplementation by HPL led to major differences in kinome activity profiles for HCC827 cells but only minor differences for A549 and H1299. In HCC827 cells, we could show increased activation of upstream receptor kinases and increased regulation of associated pathways like MAPK, Ras, and PI3K-Akt. Phenotypically, we could demonstrate that HPL treatment led to significant morphological changes in HCC827 cells, but not in A549 and H1299 cells. In HCC827 cells, the HPL-cultured cells showed cellular clustering, actin reorganization through accumulation at the edges and enhanced edge thickness. The presence of other cytokines and growth factors in HPL that are absent in FBS could explain the increase in a more complex three-dimensional growth as seen in HPL-treated cells (Figure 11).

Further molecular mechanisms underlying the observed phenotypic changes remain to be elucidated. A thorough investigation of the exact composition of the natural products HPL compared to FBS might shed more light into their specific differences, especially through the well documented batch-to-batch variability of human platelet lysate depending on donor pool, platelet preparation, and fibrinogen depletion procedure. However, both FBS and HPL present undefined biological materials with batch variability and without international standardized manufacturing. Regarding HPL, batches vary through different production techniques, which is why a change of manufacturers should be avoided and if necessary, the same manufacturing technique should be used (Immalaraju et al., 2025). Besides batch variability, contamination with non-enveloped viruses or prions is a possible source of contamination in both HPL and FBS. As the source of HPL are donor centers with routine donor screening on specific viruses, the chance of viral contamination remains low. Beyond that, specific virus inactivated HPL is available on the market. Regarding risk of prion contamination, testing for prions remains technically complicated and cost intensive. Following this, we recommend the use of virus inactivated GMP grade HPL and handle it with care to avoid contamination of the experimenters with prions. If working with prion-sensitive samples, possible infections should be checked. Lastly, the scientifically best option would be a chemically defined medium, avoiding sources of viral or prion contamination and eliminating batch variability. Besides these advantages, disadvantages are cost, especially for small volumes of media, as all growth factors and nutrients have to be ordered separately and optimization for different cell lines have to be undertaken, even though first universal chemically defined media like OUR medium exist (Rafnsdóttir et al., 2023). Concluding that, for smaller research labs FBS and HPL will still be the first choice and standardized manufactured HPL therefore represents a great possibility to avoid animal suffering and environmental harm while on the same side representing more human-like conditions. Regarding production of pharmaceuticals in cell culture as well as research labs with greater budget, chemically defined serum free media should be the media of choice, representing the scientifically best option.

Expanding our demonstrated approach towards analyzing further differences regarding HPL and FBS and employing a broader range of cell lines will provide a more comprehensive understanding of HPL’s potential towards cellular models in adherent *in vitro* experiments. Our findings provide a solid basis to further explore the use of HPL for potential human-like growth conditions of HCC827 cells. On the other hand, our results show that background signaling pathway activation remains higher in HPL-treated HCC827 cells and multiple further cellular parameters like viability, cellular marker proteins, phenotype and growth rate differ significantly from FBS. Therefore, HPL is not suited as a serum replacement for high-throughput methods using HCC827 cells. Nevertheless, using HPL for HCC827 cells offers new possibilities to grow these cells in an animal-free human-like media and presenting a more realistic *in vivo* growth state with more complex 3D-like aggregates instead of monolayers in cell culture flasks. Furthermore, our research points to superior characteristics of HPL promoting three-dimensional growth. Regarding promotion of spheroid growth, HPL shows potential through increased growth and more uniform shape, beneficial for facilitating more comparable experimental conditions. On the same side, further research on more spheroid parameters like parts of living and dead or necrotic cells and proteomics could further characterize advantages or disadvantages of HPL compared to FBS media supplementation besides increased uniformity. In general, we recommend thorough characterization of cell lines side-by-side upon HPL and FBS supplementation to decide which condition is suitable for an envisioned *in vitro* model and specific biological questions.

## Data Availability

The original contributions presented in the study are included in the article/Supplementary Material, further inquiries can be directed to the corresponding authors.
