# Supplementary material for: Phenotypic transformation of HCC827 cells revealed by evaluation of human platelet lysate as a sustainable fetal bovine serum replacement
Source: Front Cell Dev Biol. 2026 May 13;14:1791121. doi: 10.3389/fcell.2026.1791121 (PMC13212309; doi:10.3389/fcell.2026.1791121)
Supplement: Supplementary file 1 [file DataSheet1.docx]

Supplementary Information

# Supplementary Data

Supplementary Material for article “Phenotypic Transformation of HCC827 Cells Revealed by Evaluation of Human Platelet Lysate as a Sustainable Fetal Bovine Serum Replacement”.

**Content:**

| SI Figures 1-7 | Bright field microscopy images of HCC827 treated with FBS, HPL or FBS spiked with TGF-β |
| --- | --- |
| SI Figures 8-9 | Full PTK and STK upstream kinase analysis of HCC827 cells in “fresh” and “lasting” conditions |
| SI Figures 10-11 | Clustered HeatMaps of peptide phosphorylation levels from PTK and STK comparing HCC827, A549 and H1299 between “fresh” and “lasting” conditions |
| SI Figure 12 | Projection of comparisons of kinase analysis in HCC827 “fresh” and “lasting” conditions on the kinome tree |
| SI Figure 13 | Graphs for quantification of TGF-β and FGF-2 by ELISA |
| SI Figures 14-16 | Raw full-length images of uncropped western blot gels and membranes |
| SI Figures 17-22 | Peptide phosphorylation changes data behind results from upstream kinase analysis for kinases with connection to integrin signaling |


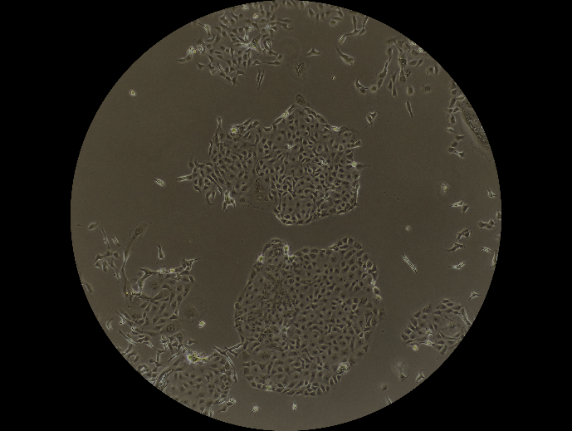


**Supplementary Figure 1.** HCC827 cells treated with 10 % FBS with 10fold magnification.


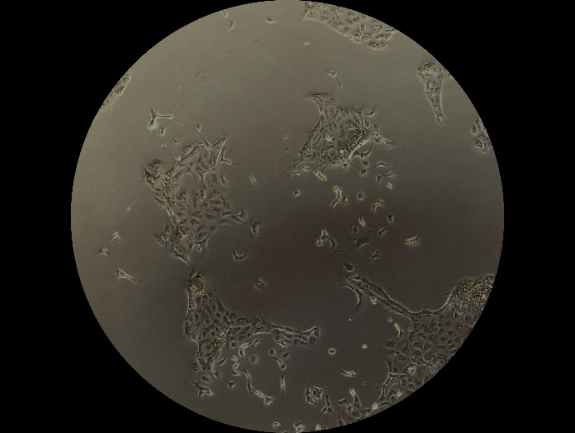


**Supplementary Figure 2.** HCC827 cells treated with 10 % HPL with 10fold magnification.


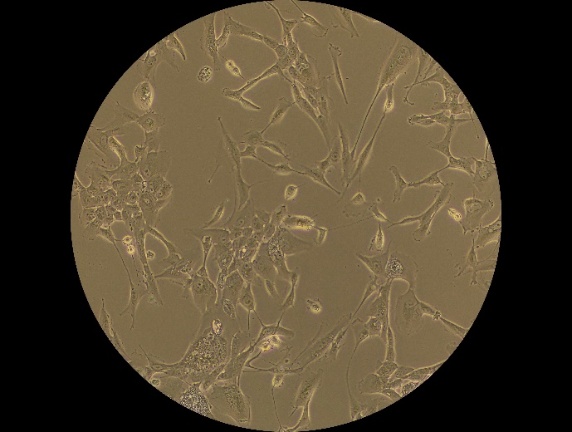


**Supplementary Figure 3.** HCC827 cells treated with 10 % FBS spiked with 100 µM TGF-β1 with 20fold magnification.


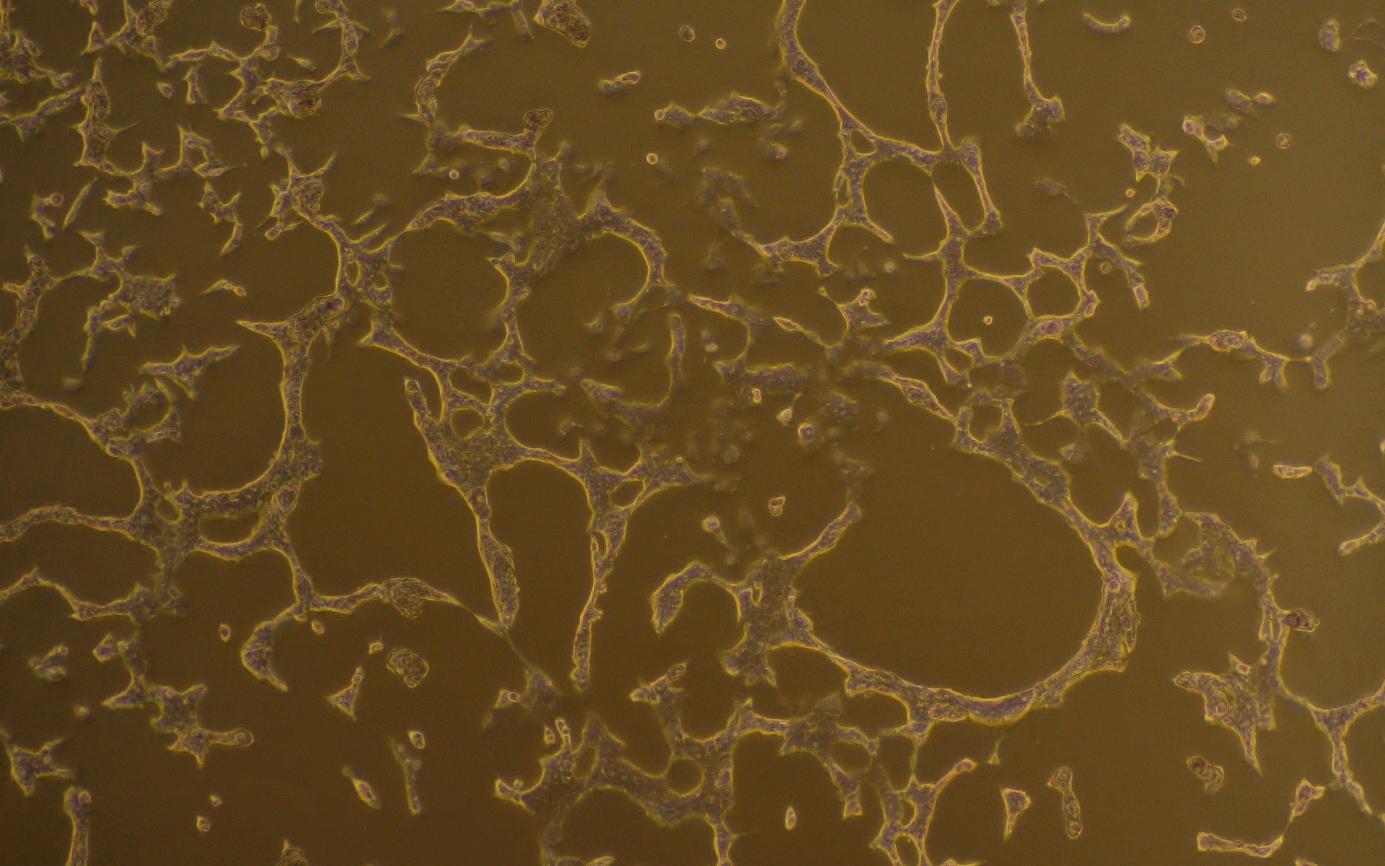

 **Supplementary Figure 4.** HCC827 cells treated with 10 % HPL with 10fold magnification


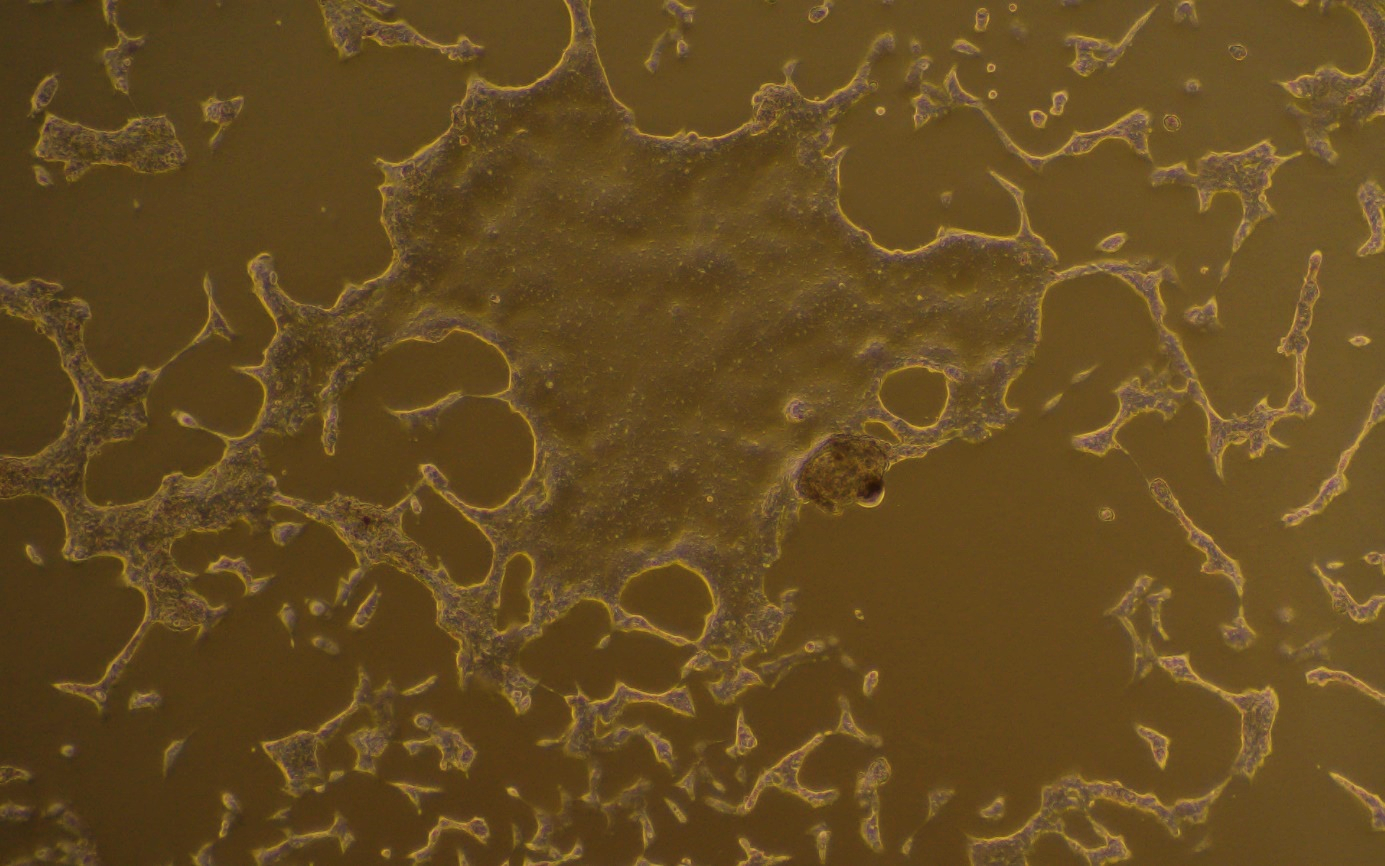
 **Supplementary Figure 5.** HCC827 cells treated with 10 % HPL with 10fold magnification.


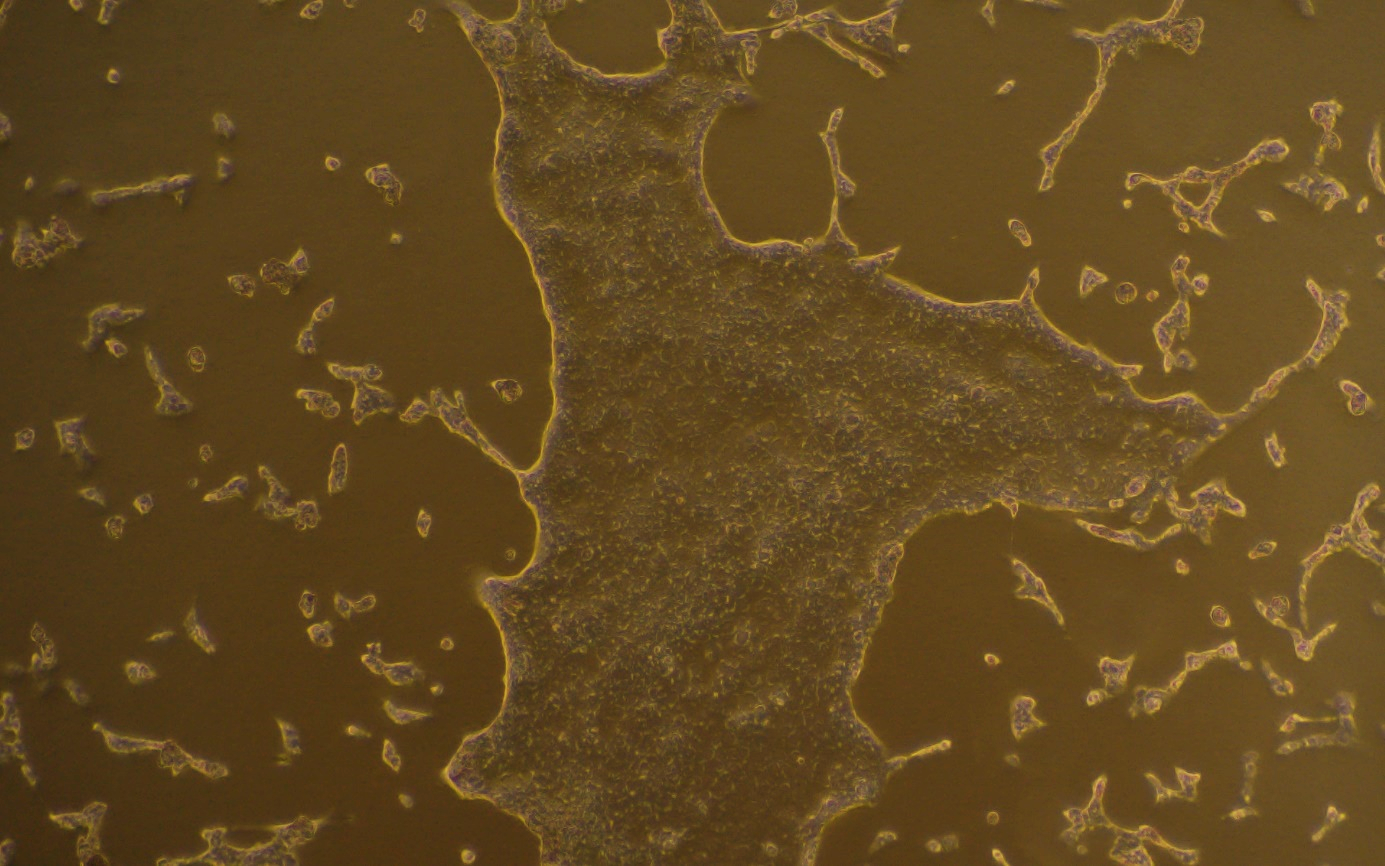
 **Supplementary Figure 6.** HCC827 cells treated with 10 % HPL with 10fold magnification.

*
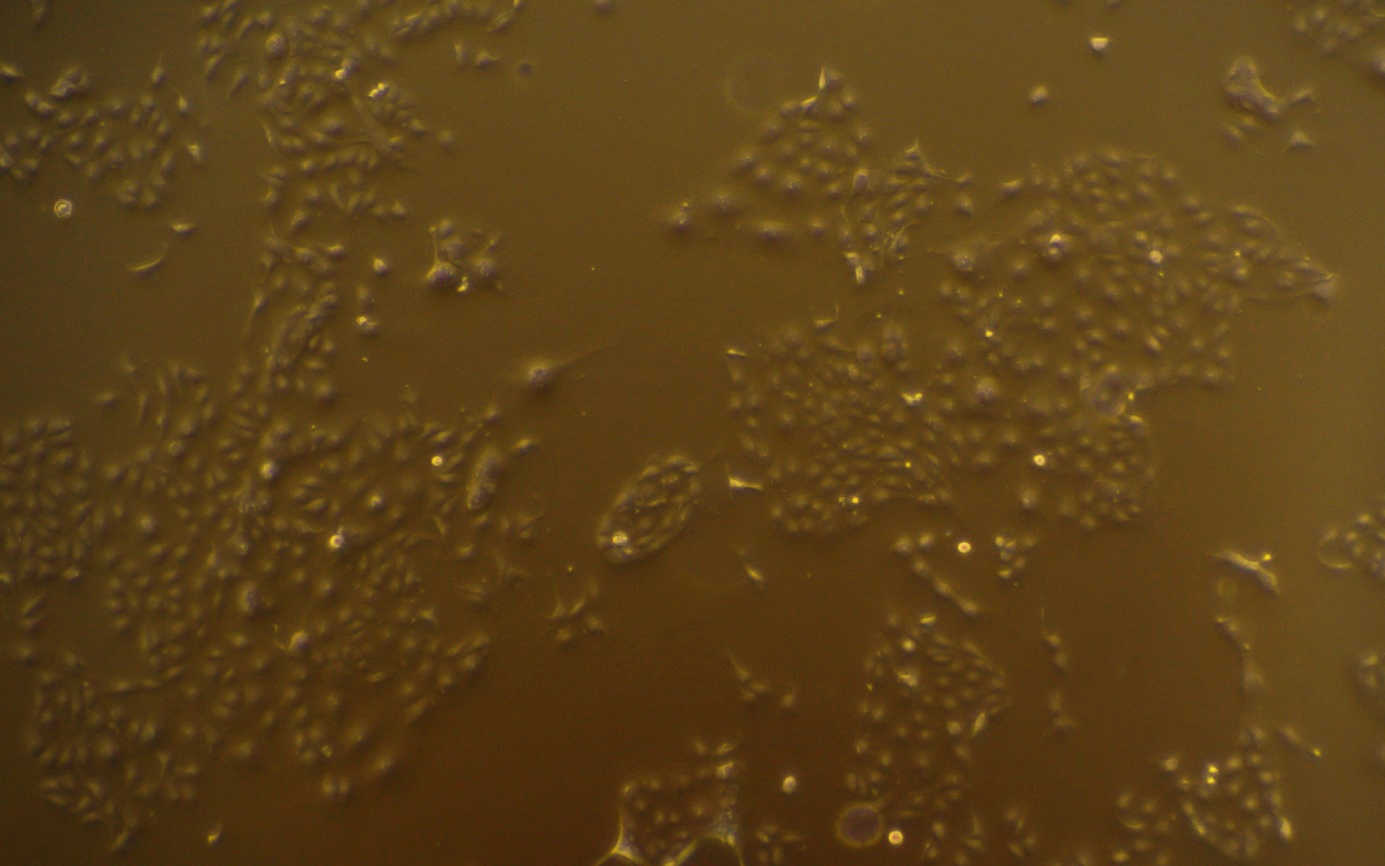
* **Supplementary Figure 7.** HCC827 cells treated with 10 % FBS with 10fold magnification.


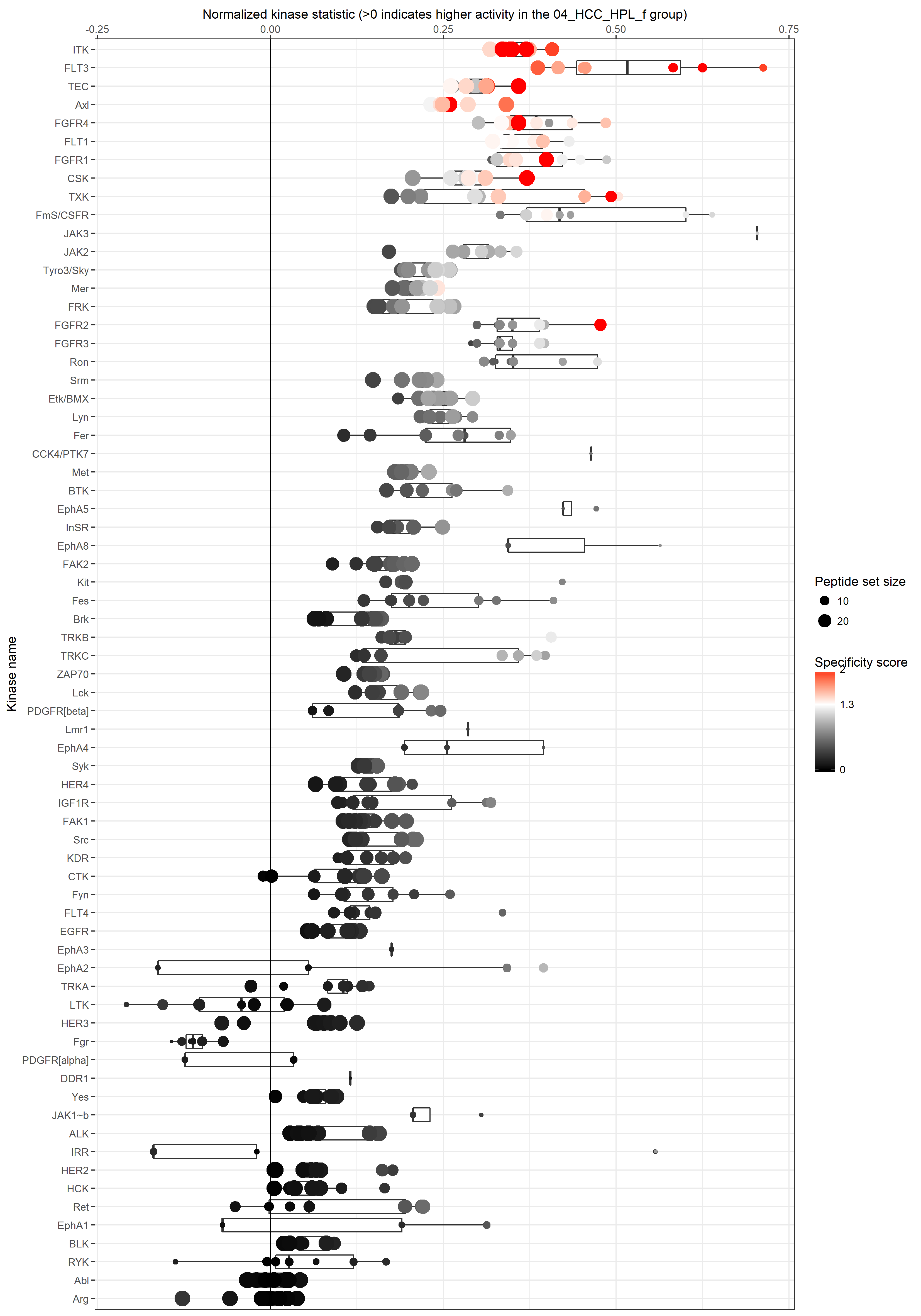


**
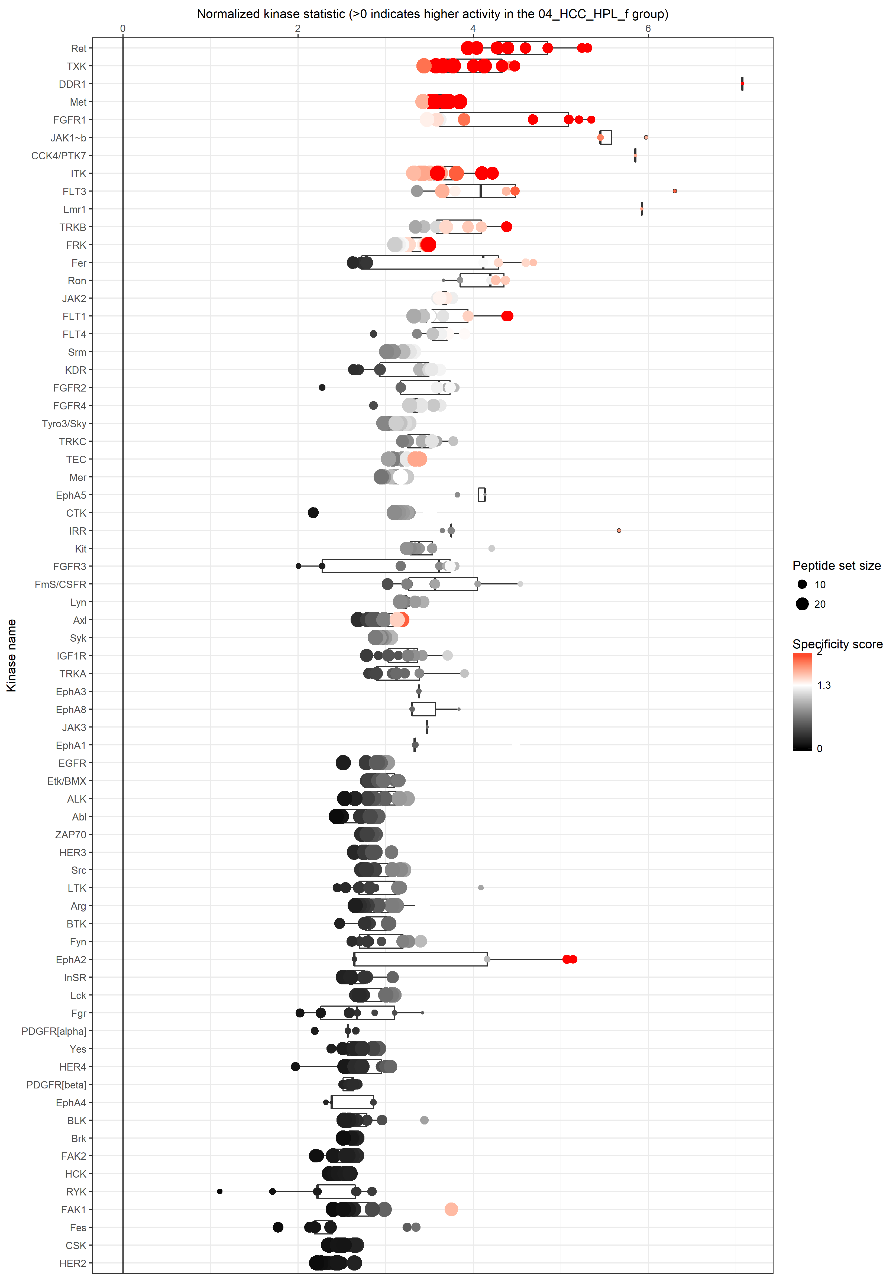

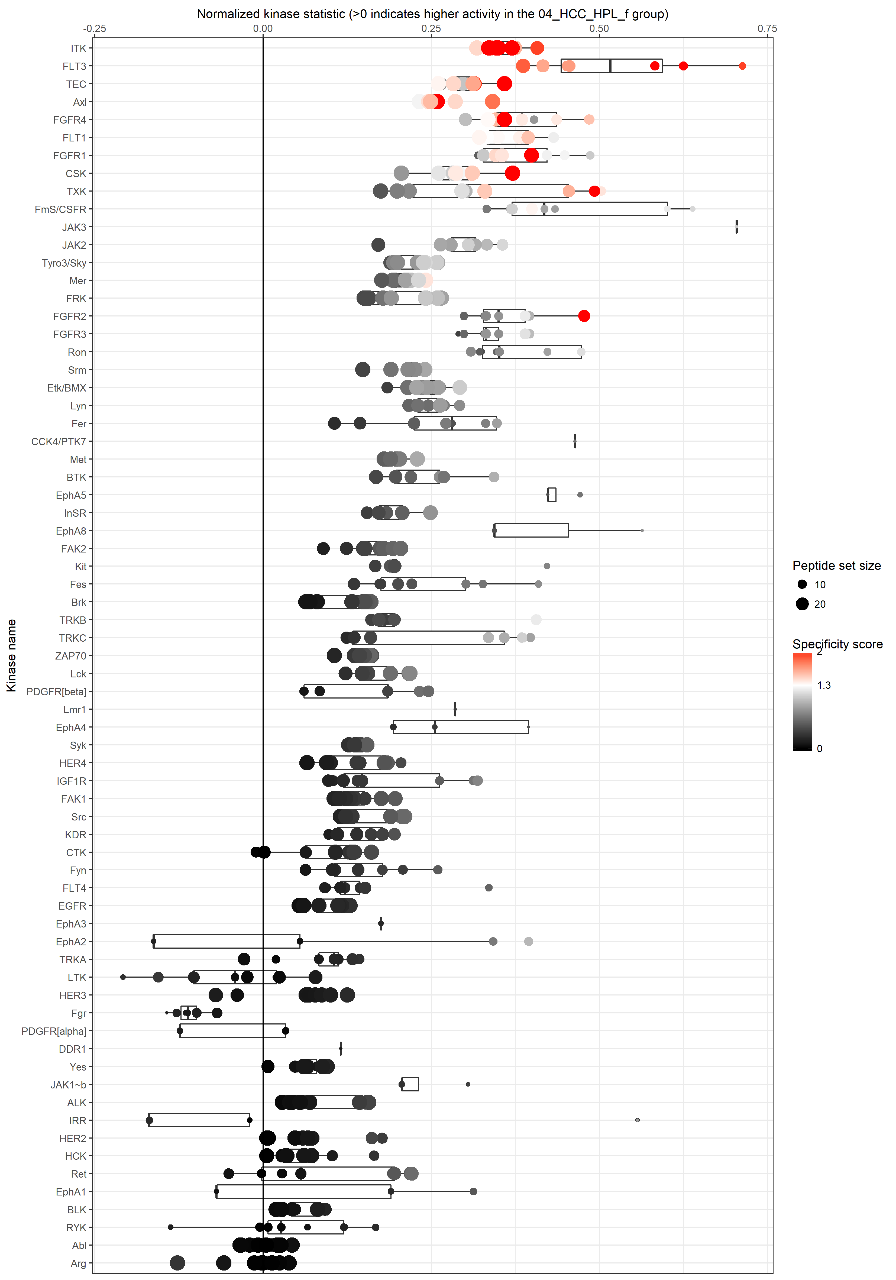
Supplementary Figure 8.** Full PTK upstream kinase analysis of HPL f vs FBS f (left) and HPL f vs HPL n (right).


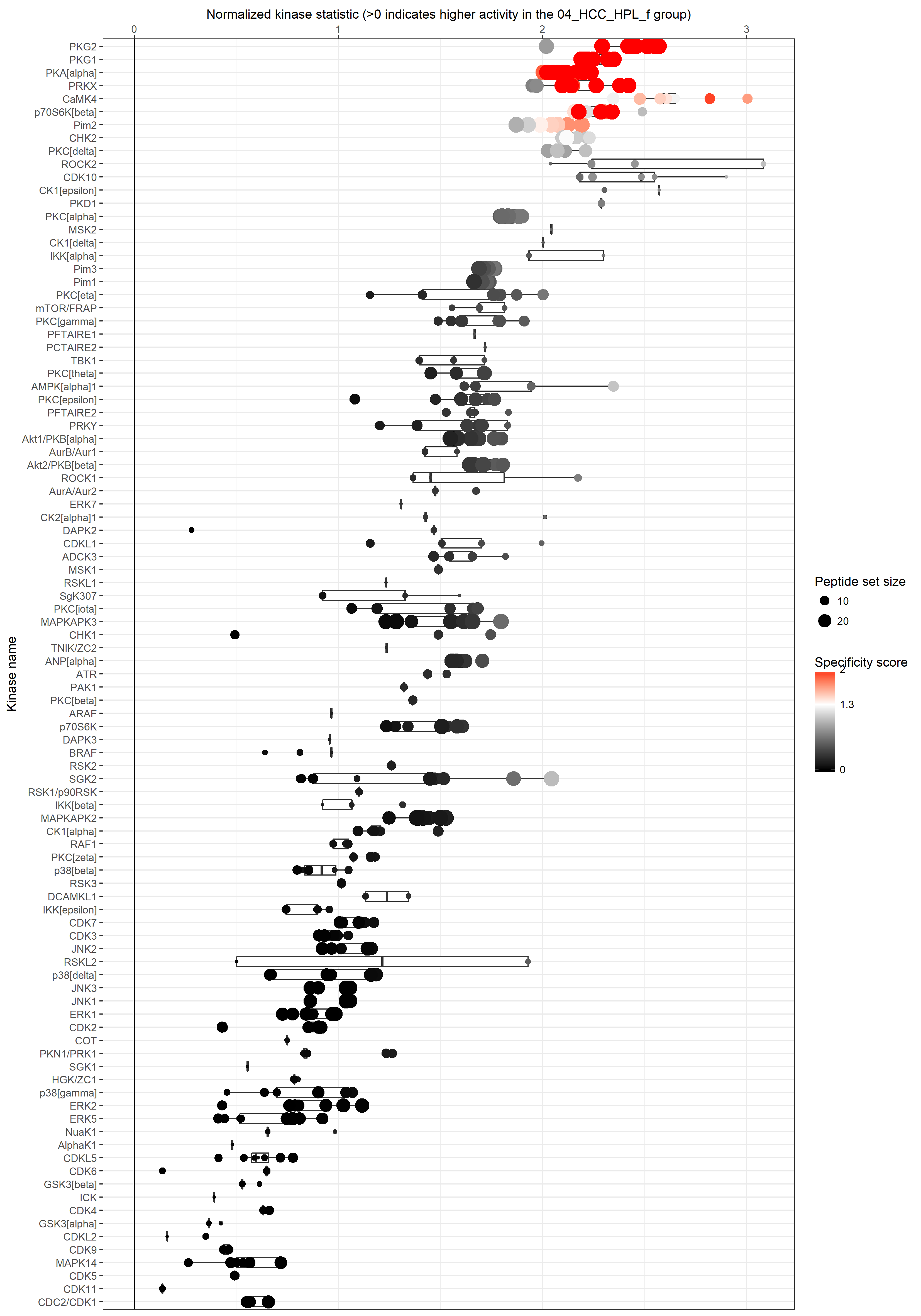


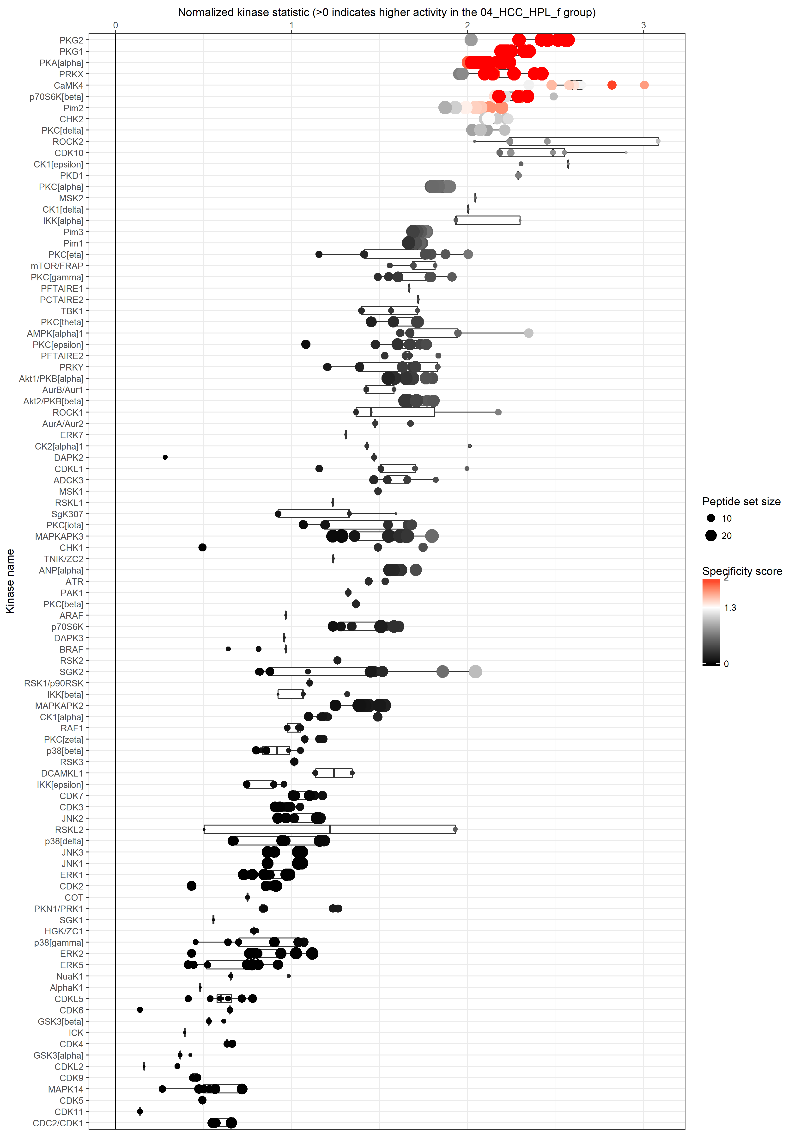

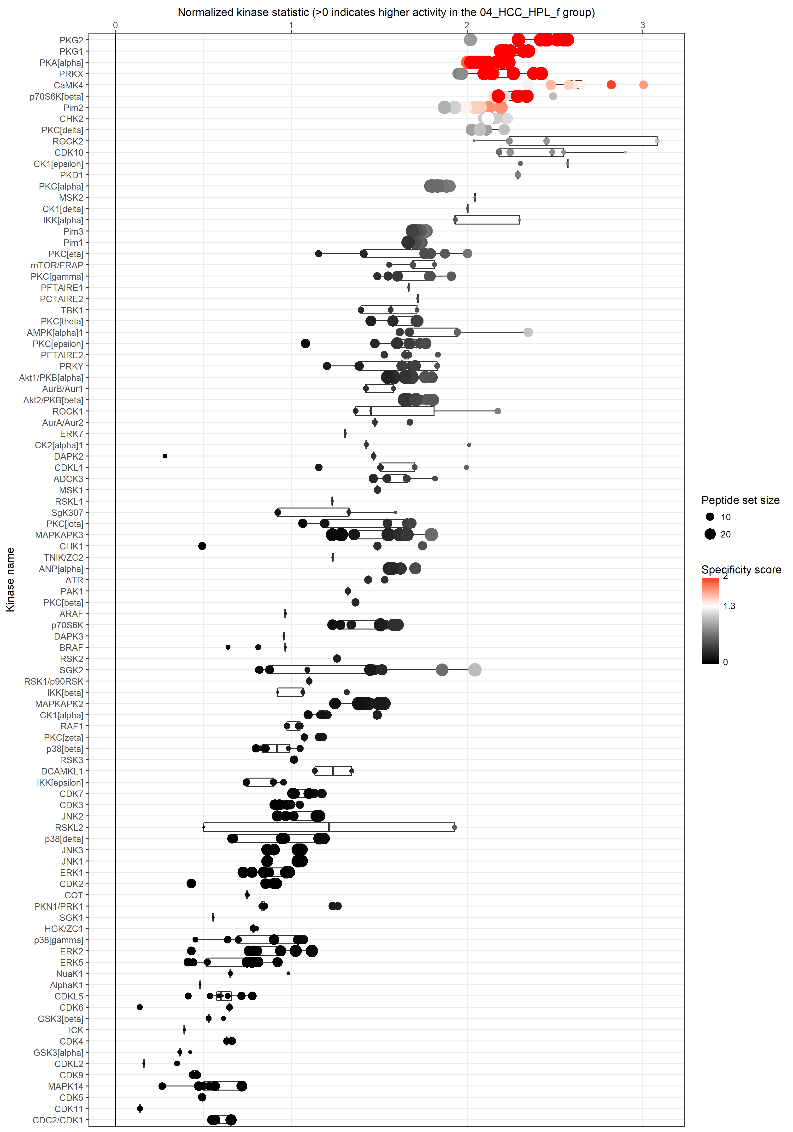


**Supplementary Figure 9.** Full STK upstream kinase analysis of HPL f vs FBS f (left) and HPL f vs HPL n (right).


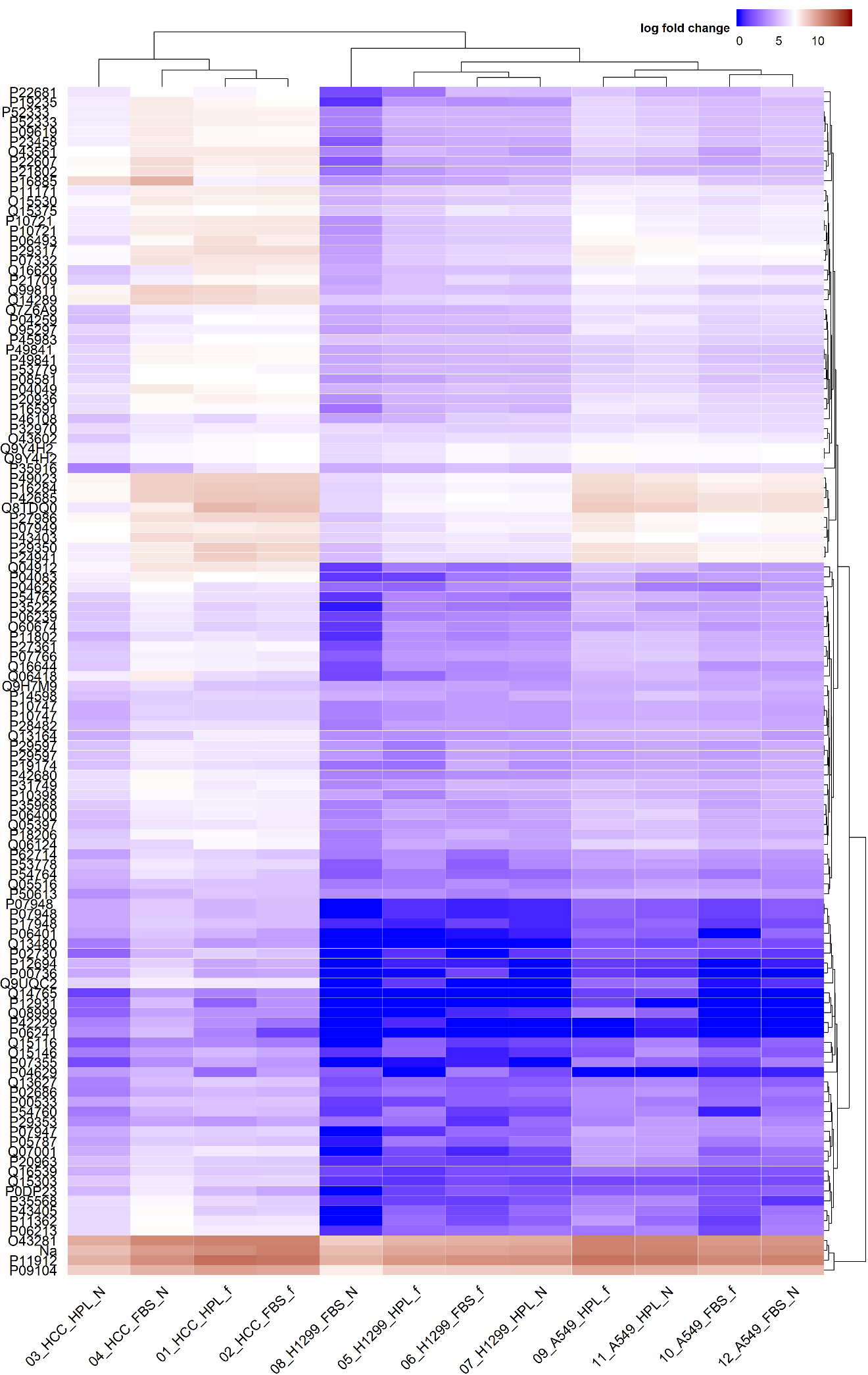


**Supplementary Figure 10.** Clustered HeatMap of peptide phosphorylation levels on PTK-chip. Descriptor f stand for “fresh” activation, while descriptor N stands for “lasting”activation”. Peptides are abbreviated by UniProt IDs.


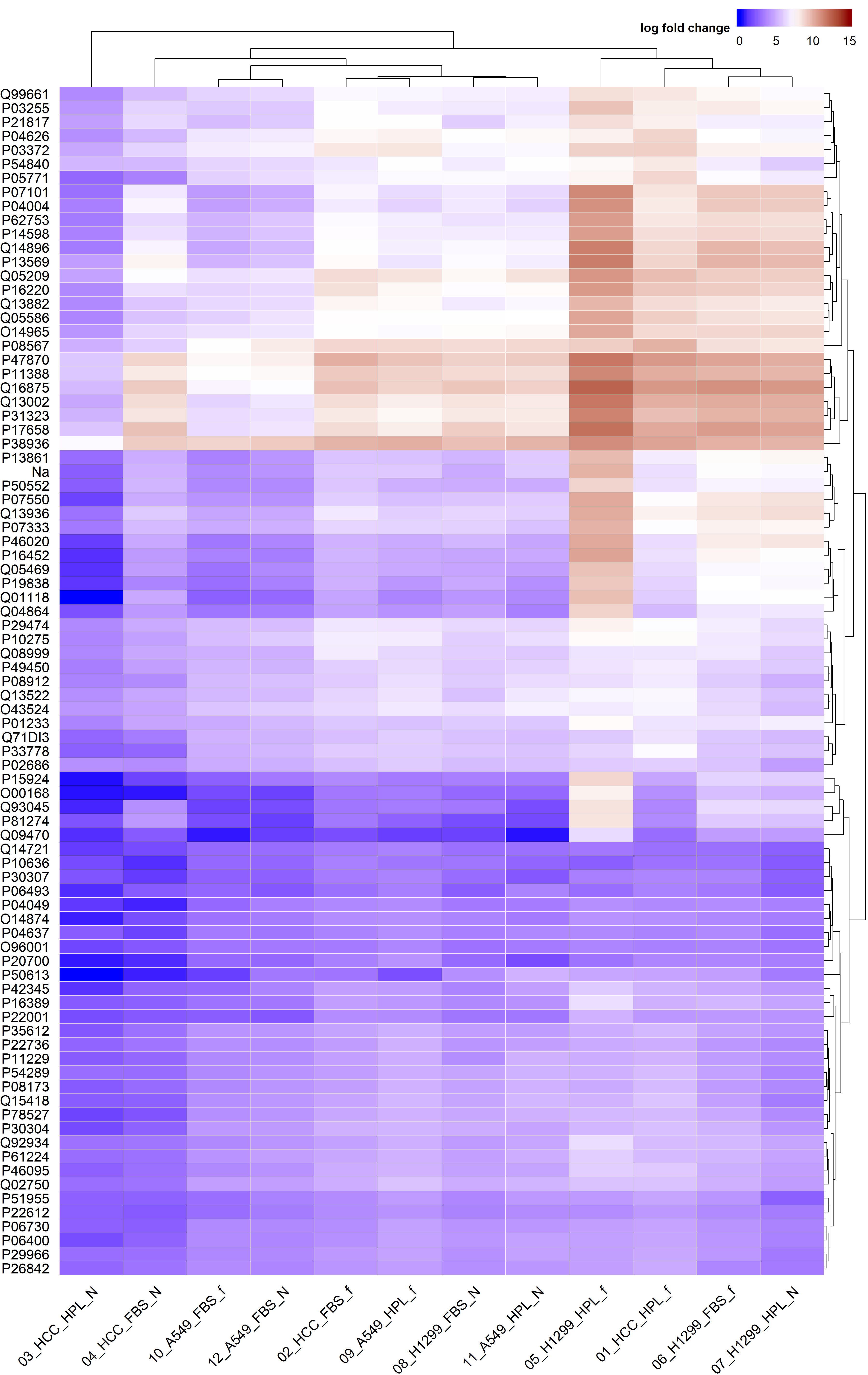


**Supplementary Figure 11.** Clustered HeatMap of peptide phosphorylation levels on STK-chip. Descriptor f stand for “fresh” activation, while descriptor N stands for “lasting”activation”. Peptides are abbreviated by UniProt IDs.
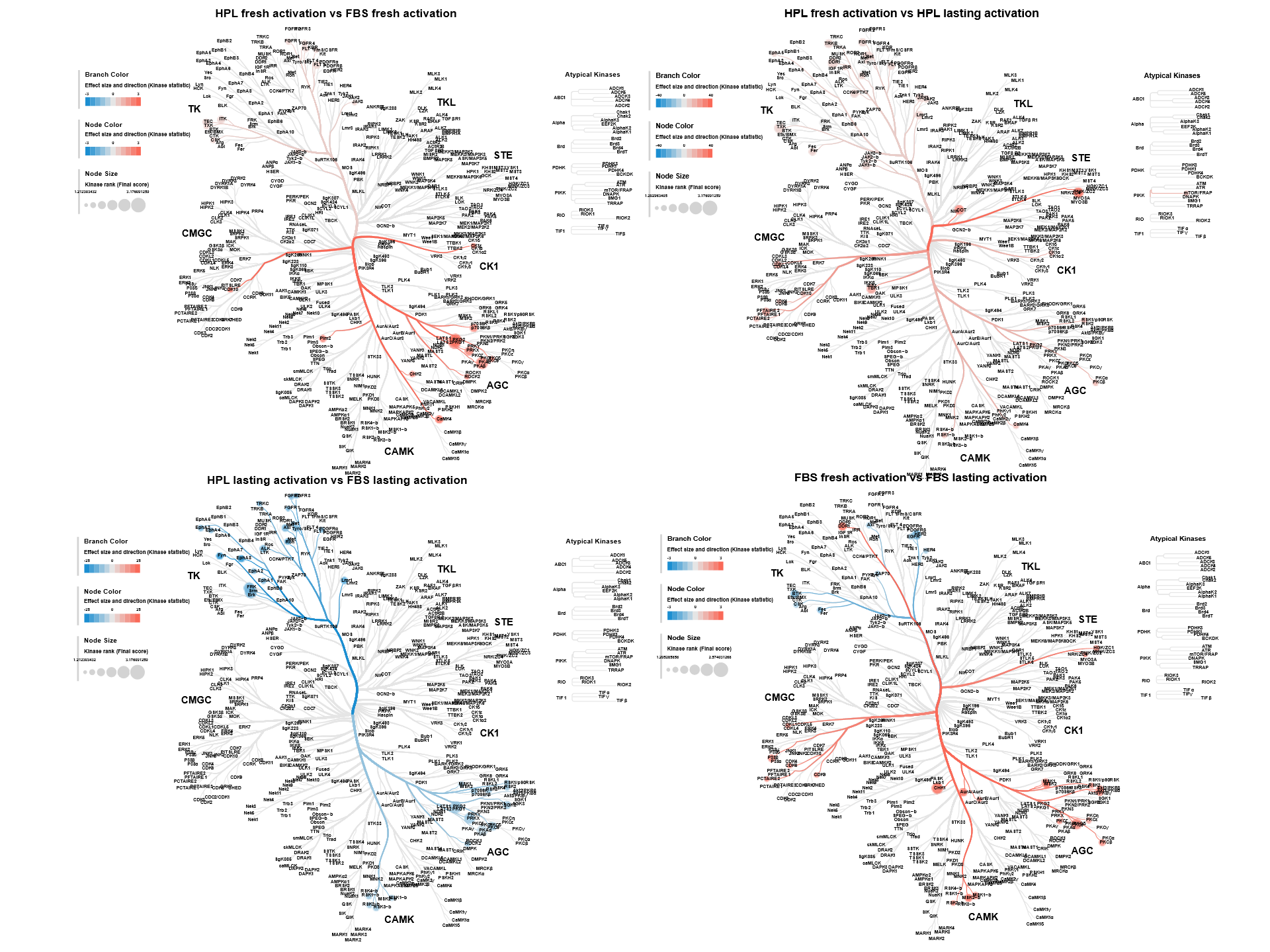


**Supplementary Figure 12.** Kinome tree pairs for comparisons HPL f vs FBS f, HPL f vs HPL n, HPL n vs FBS n and FBS f vs FBS n in HCC827 cells using CORAL (Metz, Kathleen S., et al. "Coral: clear and customizable visualization of human kinome data." Cell systems 7.3 (2018): 347-350.).


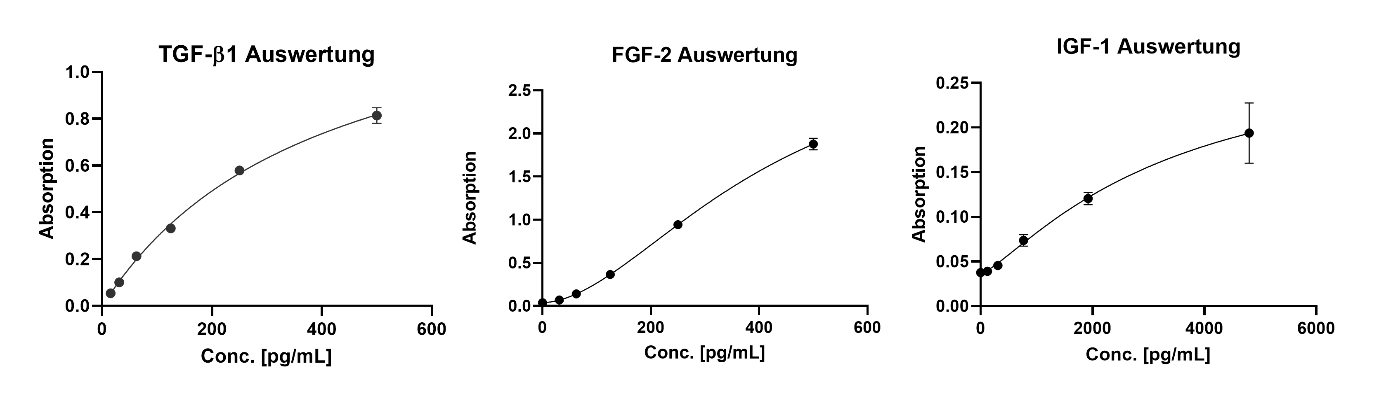


**Supplementary Figure 13.** Curve fit for ELISA assays standard dilution series.


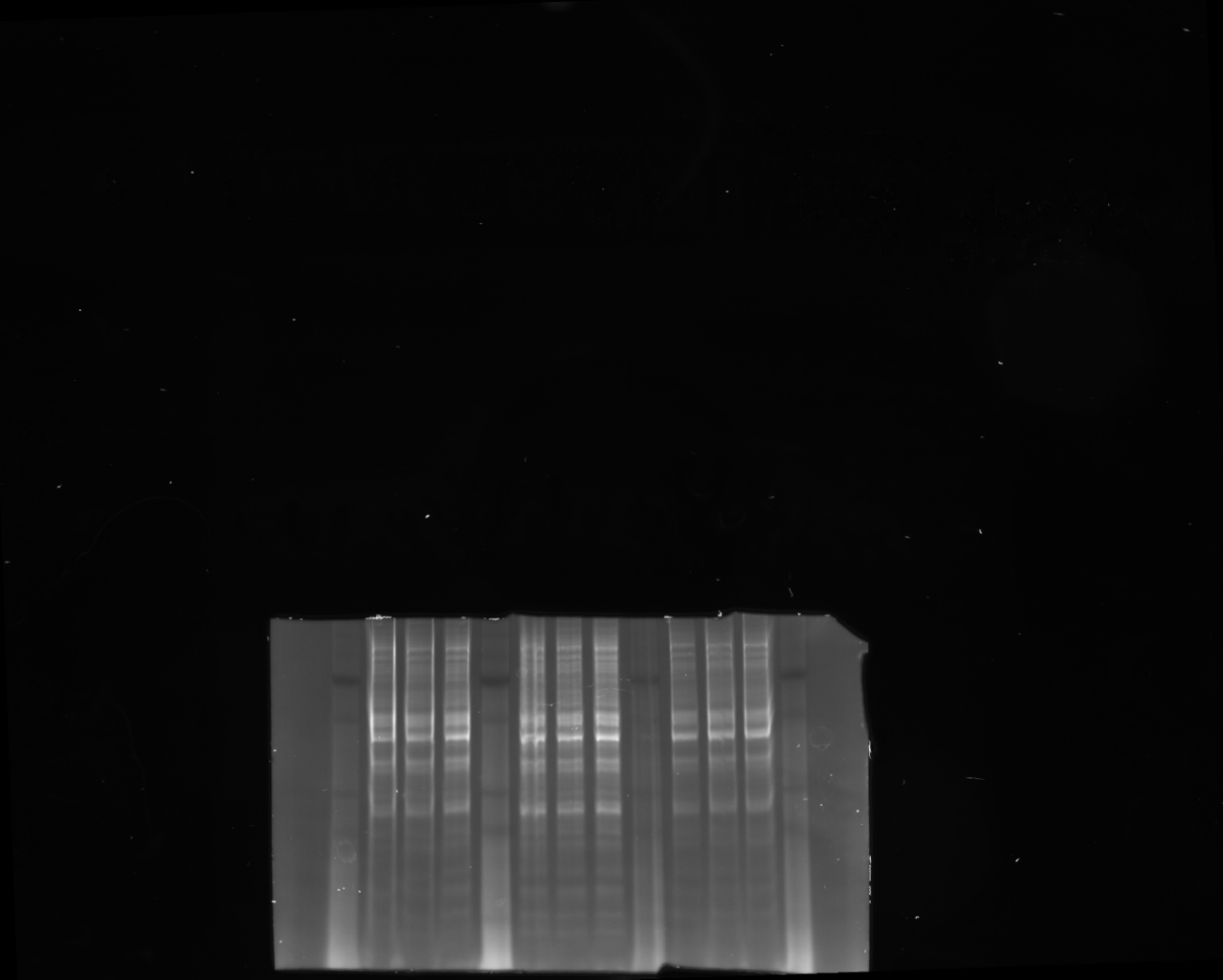

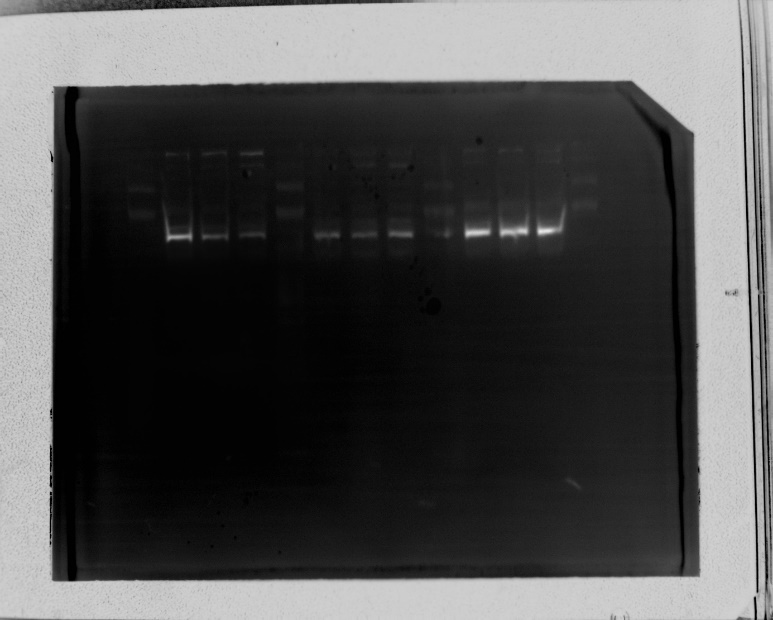


**Supplementary Figure 14.** Raw images of TCE-stained full length and uncropped gel (left) and membrane with antibody against Vimentin (right), referring to figure 9 in the manuscript.


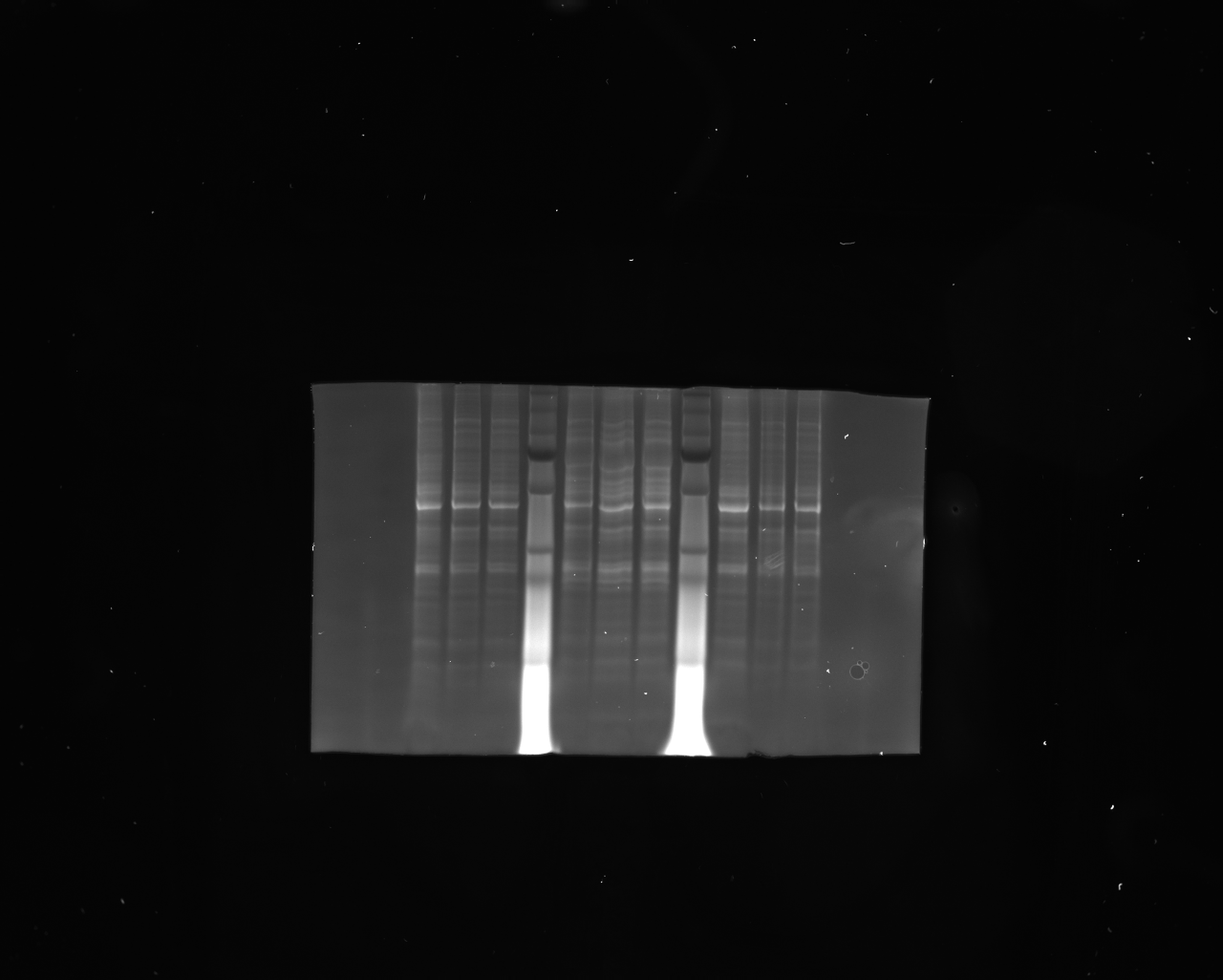

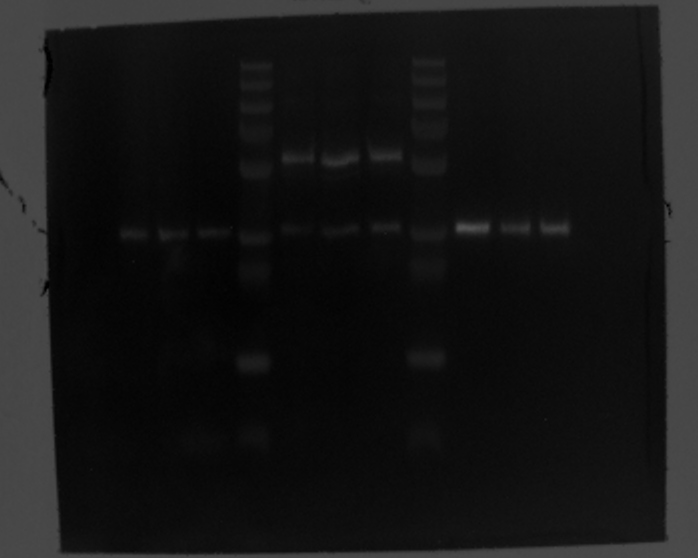


**Supplementary Figure 15.** Raw images of TCE-stained full length and uncropped gel (left) and membrane with antibody against SNAIL (right), referring to figure 9 in the manuscript.


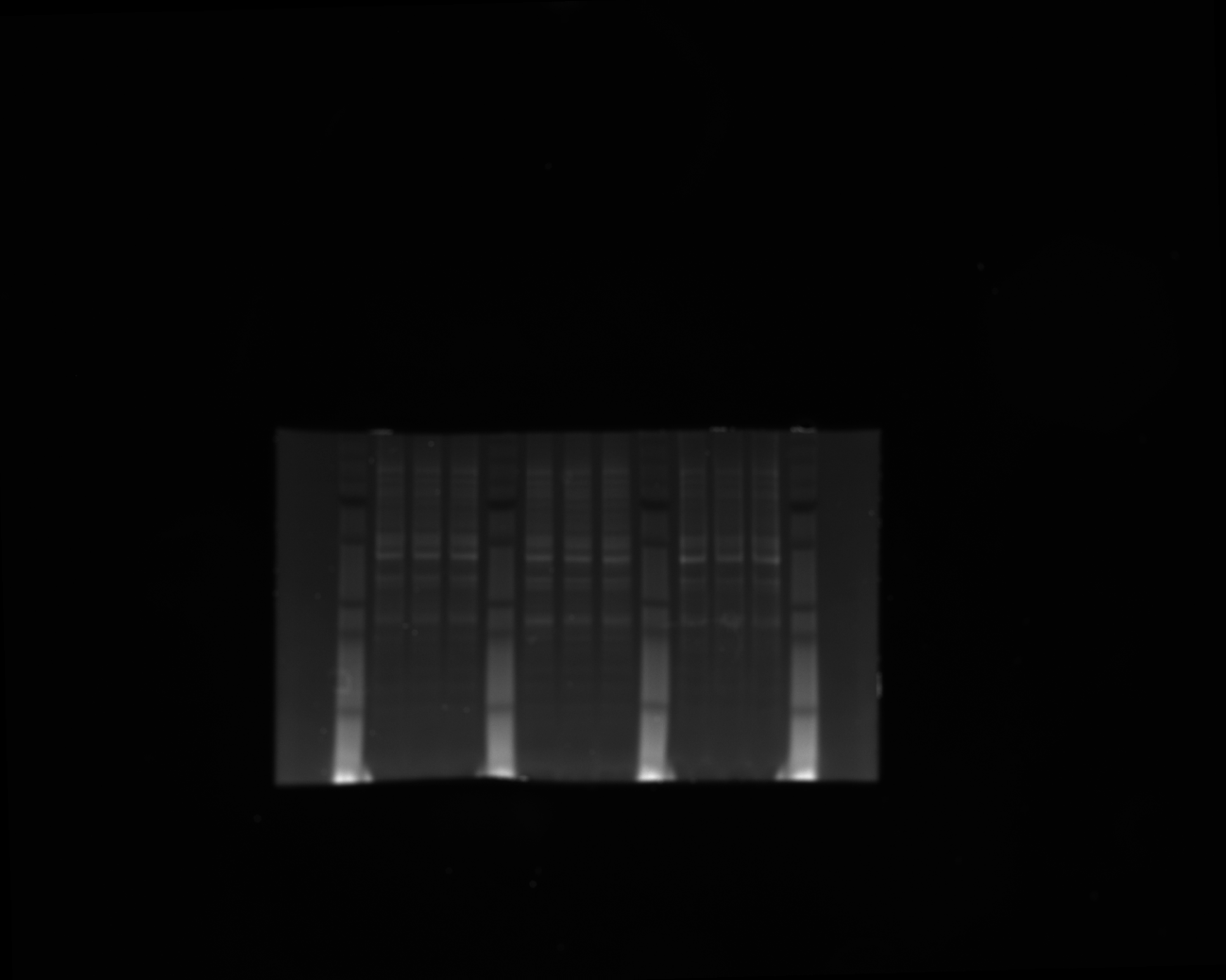

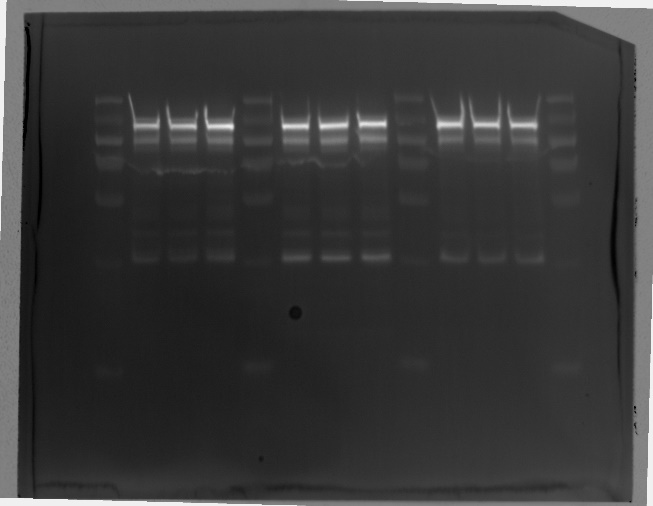


**Supplementary Figure 16.** Raw images of TCE-stained full length and uncropped gel (left) and membrane with antibody against E-Cadherin (right), referring to figure 9 in the manuscript.


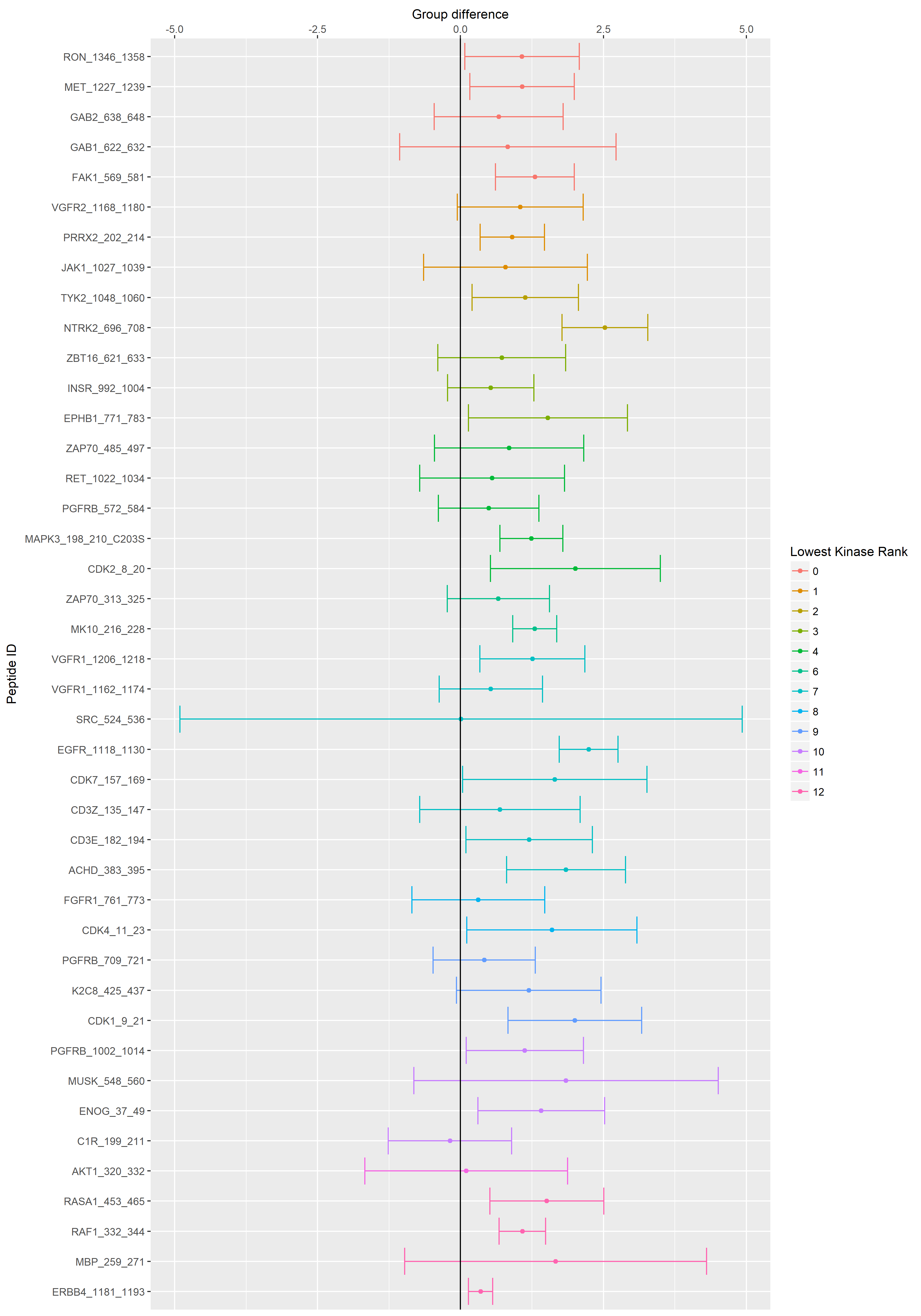


**Supplementary Figure 17.** Background data of PTK upstream kinase analysis for MET in HPL f vs HPL n showing peptide phosphorylation levels of peptides addressed by specific kinase. Kinase rank is based on credibility of data sets. Lower kinase ranks means higher credibility. Credibility increases if background data is from (multiple) in vivo or in vitro data and is lower if it is from single in vitro or in silico data.


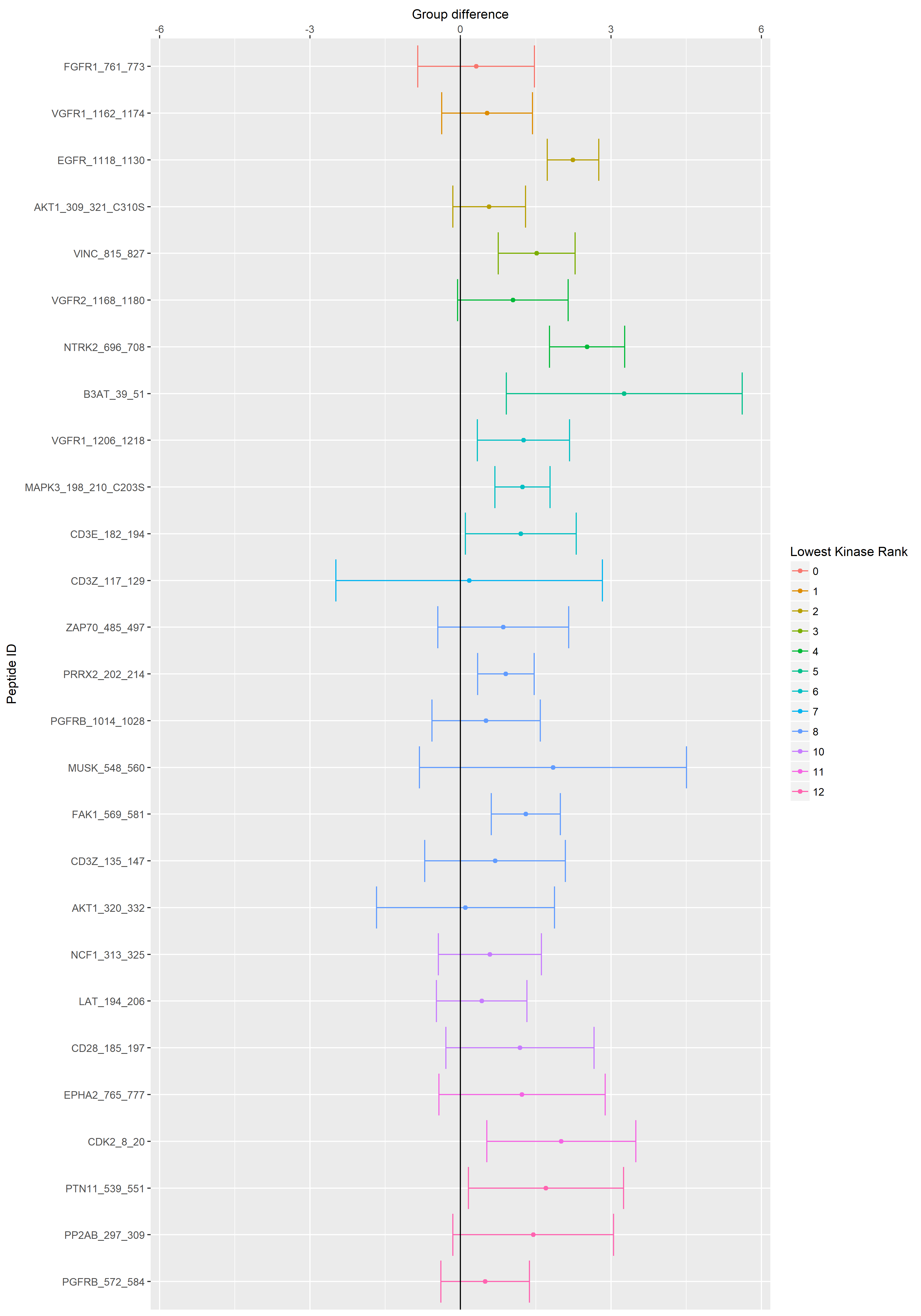


**Supplementary Figure 18.** Background data of PTK upstream kinase analysis for FGFR1 in HPL f vs HPL n showing peptide phosphorylation levels of peptides addressed by specific kinase. Kinase rank is based on credibility of data sets. Lower kinase ranks means higher credibility. Credibility increases if background data is from (multiple) in vivo or in vitro data and is lower if it is from single in vitro or in silico data.


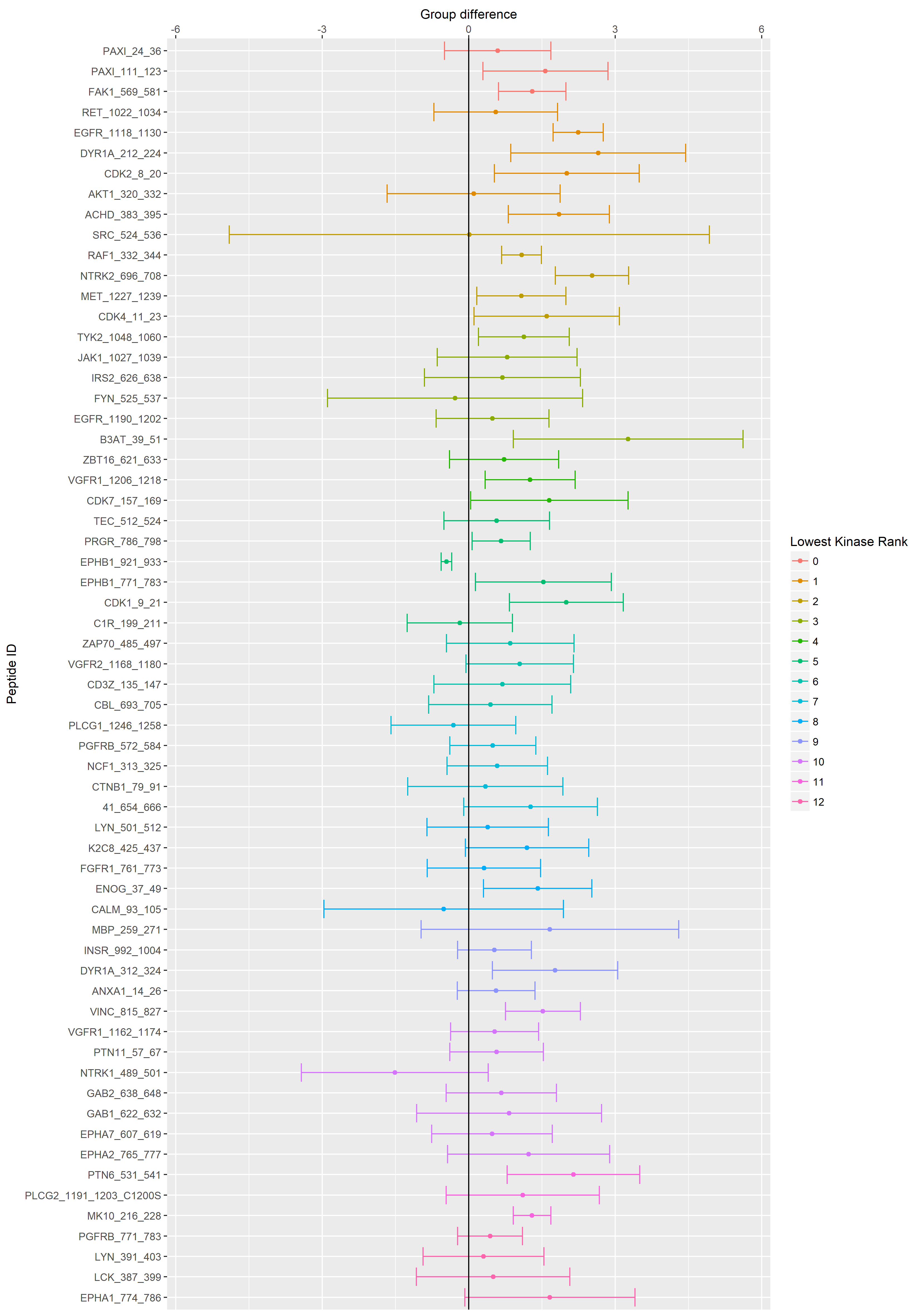


**Supplementary Figure 19.** Background data of PTK upstream kinase analysis for FAK1 in HPL f vs HPL n showing peptide phosphorylation levels of peptides addressed by specific kinase. Kinase rank is based on credibility of data sets. Lower kinase ranks means higher credibility. Credibility increases if background data is from (multiple) in vivo or in vitro data and is lower if it is from single in vitro or in silico data.


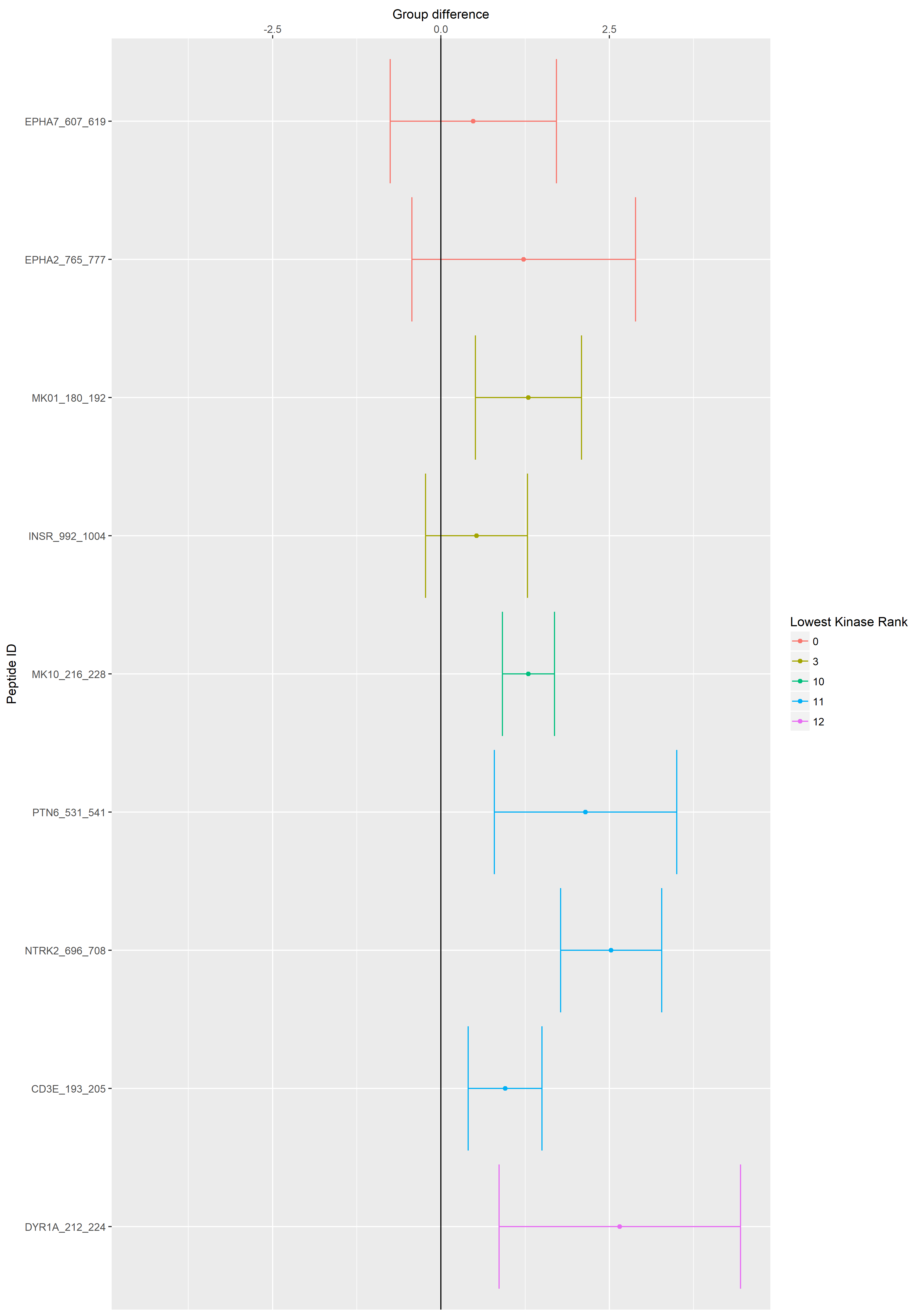


**Supplementary Figure 20.** Background data of PTK upstream kinase analysis for EphA2 in HPL f vs HPL n showing peptide phosphorylation levels of peptides addressed by specific kinase. Kinase rank is based on credibility of data sets. Lower kinase ranks means higher credibility. Credibility increases if background data is from (multiple) in vivo or in vitro data and is lower if it is from single in vitro or in silico data.


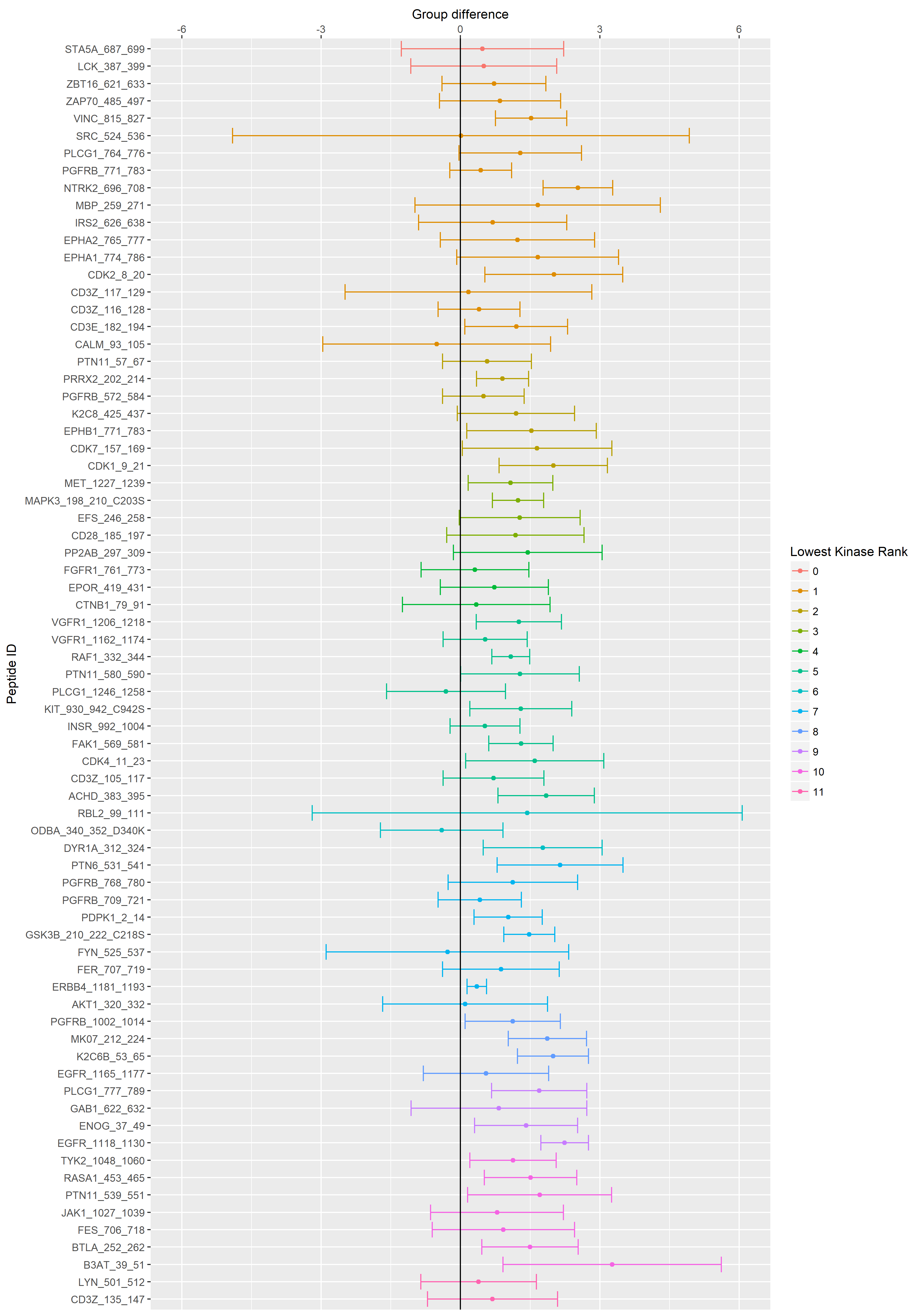


**Supplementary Figure 21.** Background data of PTK upstream kinase analysis for Axl in HPL f vs HPL n showing peptide phosphorylation levels of peptides addressed by specific kinase. Kinase rank is based on credibility of data sets. Lower kinase ranks means higher credibility. Credibility increases if background data is from (multiple) in vivo or in vitro data and is lower if it is from single in vitro or in silico data.


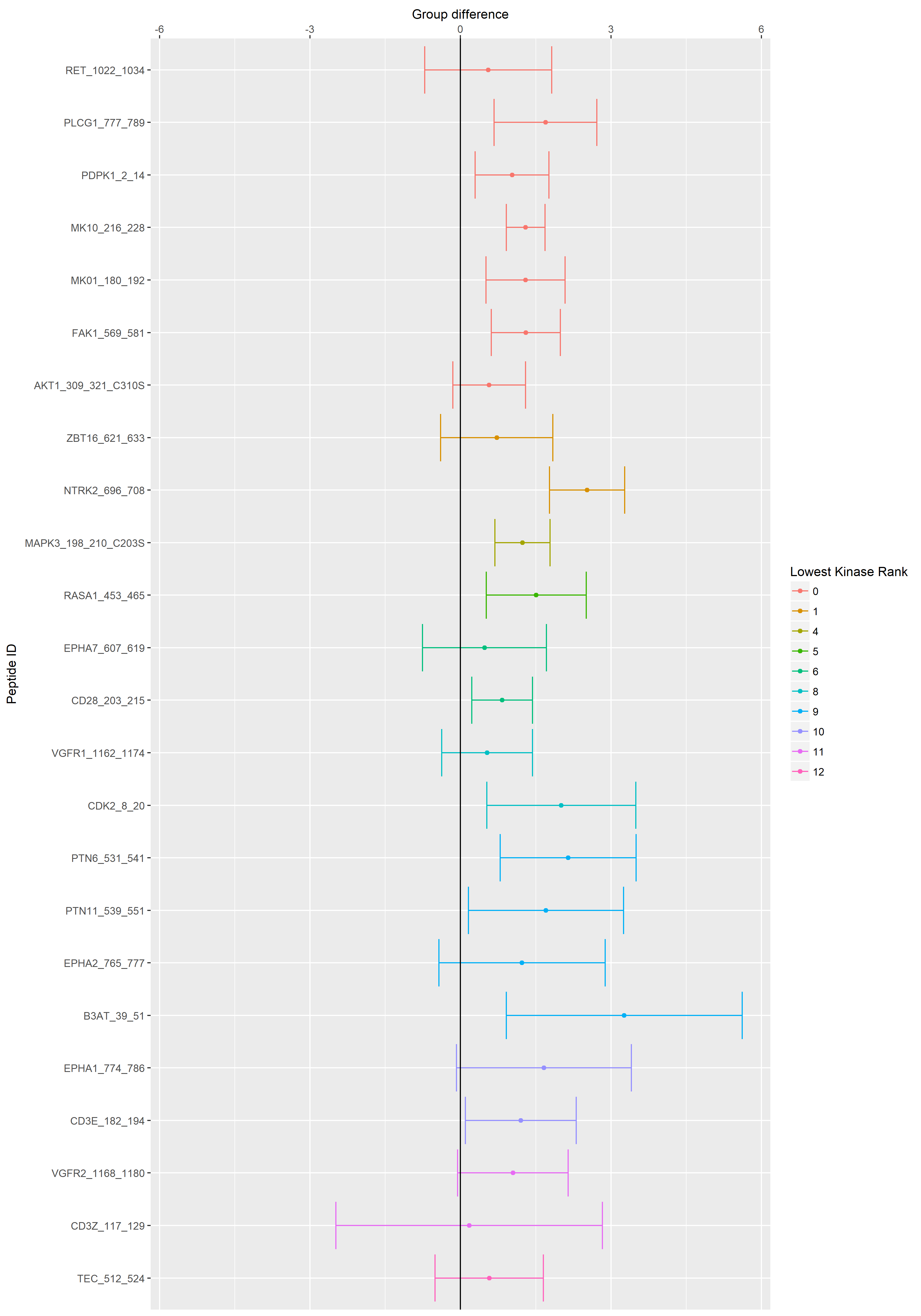
 **Supplementary Figure 22.** Background data of PTK upstream kinase analysis for Ret in HPL f vs HPL n showing peptide phosphorylation levels of peptides addressed by specific kinase. Kinase rank is based on credibility of data sets. Lower kinase ranks means higher credibility. Credibility increases if background data is from (multiple) in vivo or in vitro data and is lower if it is from single in vitro or in silico data.
